# Mapping Misconceptions in Neuroendocrine Tumor Nomenclature: A Scoping Review of National Database Studies and Clinical Implications

**DOI:** 10.3390/cancers18152508

**Published:** 2026-08-05

**Authors:** Theo F. Hanson, Margaret Hua, Jorge Zarate Rodriguez, Lauren H. Yaeger, Shreya Rao Chilukuri, Nikolaos A. Trikalinos, Chet W. Hammill

**Affiliations:** 1 School of Medicine, University College Dublin, D04 V1W8 Dublin, Ireland; 2 Washington University School of Medicine, St. Louis, MO 63110, USA; 3 Department of Surgery, Washington University School of Medicine, St. Louis, MO 63110, USA; 4 Bernard Becker Medical Library, Washington University School of Medicine, St. Louis, MO 63110, USA; 5 Division of Medical Oncology, Department of Medicine, Washington University School of Medicine, St. Louis, MO 63110, USA; ntrikalinos@wustl.edu; 6 The Oregon Clinic Center for Advanced Surgery–East, Portland, OR 97213, USA

**Keywords:** neuroendocrine tumors, neuroendocrine neoplasms, nomenclature, WHO classification, SEER program, National Cancer Database, scoping review

## Abstract

In 2010, the World Health Organization (WHO) standardized neuroendocrine neoplasm terminology, dividing these tumors by differentiation into well-differentiated neuroendocrine tumors and poorly differentiated neuroendocrine carcinomas. Registries such as the Surveillance, Epidemiology, and End Results (SEER) program and the National Cancer Database (NCDB) store legacy codes predating this framework, raising concern that registry-based research carries nomenclature errors into the clinical literature. We performed a scoping review of research published in 2012–2022 that used SEER and/or the NCDB to study gastroenteropancreatic neuroendocrine tumors, charting 170 articles (from 1079 citations) against the 2010 classification, with forward citation analysis (OpenAlex, Semantic Scholar) measuring downstream citation exposure. Of 141 assessable studies, 88% (*n* = 124) applied the nomenclature inaccurately: 82.3% included poorly or undifferentiated neoplasms within neuroendocrine tumor cohorts (mean 18.7% of the cohort) and 9.9% conflated database differentiation grade with WHO proliferation grade; appropriate usage did not improve over time. These discordant studies accumulated 7323 citations across 5145 works, with 87.9% cited by at least one review (1075 distinct reviews) and six cited by major guidelines (NCCN, ESMO, ENETS). Across research published from 2012 to 2022, nomenclature discordance was widespread, persistent throughout the study period, and present in studies frequently cited by the secondary and guideline literature. Because these cohorts incorporate more aggressive and poorly or undifferentiated neoplasms, their aggregate outcome estimates are likely biased toward a poorer prognosis, potentially overstating the aggressiveness of well-differentiated neuroendocrine tumors, particularly for prognostic estimates; this review characterized cohort composition rather than quantifying the effect on any individual study’s outcomes, and whether this bias has, in turn, affected clinical decision-making was not assessed here and remains a hypothesis for future work. Journals and guideline panels should require explicit alignment with current WHO definitions for registry-based studies.

## Simple Summary

Neuroendocrine neoplasms are a group of cancers that range from slow-growing to highly aggressive. In 2010, the World Health Organization set clear rules for naming and grading them, separating slow-growing neuroendocrine tumors from a far more dangerous form called neuroendocrine carcinoma. Many influential studies draw on large national cancer registries that still use older codes, so it is unclear how reliably those studies follow the modern naming rules. We reviewed 170 such studies and found that the great majority mixed aggressive carcinomas into groups labeled as milder tumors, which can make these cancers appear more aggressive than they truly are. We also showed that these flawed studies are heavily cited by later reviews and treatment guidelines. These misleading survival estimates could, in turn, encourage over-treatment of patients with slow-growing disease, although our review examined how the terminology is used and cited, not patients’ treatment or outcomes. We recommend that journals require studies using cancer registries to state how they align with current naming standards.

## 1. Introduction

Our understanding of neuroendocrine neoplasms has evolved significantly over the past 100 years, in terms of both their origin and their clinical behavior [1]. Initially mischaracterized by Siegfried Oberndorfer as benign and inconsequential [1,2], we now understand that they exhibit a spectrum of aggressiveness ranging from slow-growing histologies to forms that closely resemble their more common epithelial counterparts [3]. Today, we know there are various ways to characterize and predict their behavior, and currently the most helpful are histological parameters, namely differentiation and proliferation markers [1].

Pathologists have long predicted the aggressiveness of a neoplasm based on how closely the tumor cells resemble “normal” tissue. Differentiation is a subjective histological evaluation that codifies this, with well-differentiated disease looking similar to background tissue and poorly differentiated neoplasms bearing little resemblance to the original healthy tissue [4]. Another predictor of aggressiveness is the rate of cell division and multiplication. Proliferation markers such as mitotic count and Ki-67 are biological indicators of these rates [5,6].

In 2010, in an attempt to standardize nomenclature, the World Health Organization (WHO) published a classification system for neuroendocrine neoplasms that divided them by differentiation into neuroendocrine tumors (well-differentiated) and neuroendocrine carcinomas (poorly differentiated) [1]. Proliferation, assessed by mitotic count or Ki-67 index, was then assigned a numerical grade within each of these categories (Table 1) [6]. Of note, under the 2010 scheme, high proliferation was treated as a feature of poorly differentiated carcinomas; a well-differentiated tumor with high proliferation had no defined category and would not be recognized until later revisions.

A series of revisions, formalized in a 2018 international consensus and adopted in the 2022 WHO classification, added a category for well-differentiated tumors with high proliferation; well-differentiated neuroendocrine tumors are now graded G1, G2, or G3 (Table 2) [7,8]. Neuroendocrine carcinomas remained poorly differentiated by definition and were further subclassified into small-cell and large-cell types [7,8]. This analysis is nonetheless anchored to the 2010 framework, because its central division of well-differentiated tumors from poorly differentiated carcinomas is unchanged in every later edition and is the distinction most relevant to how these neoplasms are recorded in national databases.

When studying neuroendocrine neoplasms, researchers commonly use large, population-based cancer registries such as the Surveillance, Epidemiology and End Results Program (SEER) and the National Cancer Database (NCDB). As nomenclature has evolved over time, discordances have developed between how data are stored in these registries and the updated classification systems. For example, per the 2010 WHO classification framework [9], neuroendocrine tumors are by definition well-differentiated. However, both the NCDB and SEER contain data on moderately, poorly, and undifferentiated neoplasms stored under neuroendocrine tumor histologic classifications. The limitations of population-based registries for neuroendocrine neoplasm epidemiology, including absent proliferation (Ki-67) data and lagging behind an evolving classification, have been recognized [10]. While it is suspected that these database limitations are carried forward into the published literature, the scale and nature of this phenomenon across the gastroenteropancreatic neuroendocrine tumor literature remains uncharacterized.

In addition, both SEER and the NCDB store information on tumor differentiation as grade 1 (well-differentiated), grade 2 (moderately differentiated), grade 3 (poorly differentiated), and grade 4 (undifferentiated). These grades are completely distinct from the grading system (G1, G2, and G3) used for neuroendocrine neoplasms, which is based on proliferation (mitotic count or Ki-67 index). By definition, a neuroendocrine tumor is well-differentiated (SEER/NCDB grade 1 or possibly grade 2) and a neuroendocrine carcinoma is poorly differentiated (SEER/NCDB grade 3 or possibly grade 4). There is concern that researchers may be assuming that a neoplasm defined as grade 1 in SEER or NCDB is equivalent to a G1 neuroendocrine neoplasm as defined by WHO. This concern is supported by the observation of published articles utilizing SEER and the NCDB reporting data on G1, G2, and G3 neuroendocrine neoplasms when proliferation data necessary for making these categorizations is not available in the databases. Of note, the NCDB and SEER have updated their coding systems, enabling up-to-date WHO neuroendocrine neoplasm nomenclature, so this concern is mostly relevant for SEER data prior to 2021 and NCDB data prior to 2018 [11,12].

Finally, the term “carcinoid” has been discouraged by the WHO since 2000 [1,3], yet it remains common in recent publications. Coined in 1907 by Siegfried Oberndorfer for intestinal tumors that appeared less aggressive than adenocarcinomas [1,2], the use of the word for gastroenteropancreatic neuroendocrine tumors has since become not merely outdated but actively misleading. It implies an indolent, uniform behavior that misrepresents a biologically heterogeneous group of tumors which have the potential to metastasize [13]. It is further confounded by “carcinoid syndrome,” a hormonal syndrome that arises in only a minority of these tumors, so that one word denotes both a tumor type and a clinical syndrome that most such tumors never produce. Well-differentiated neoplasms of the digestive tract are therefore now designated neuroendocrine tumors [8], and even in the lung, where “carcinoid” has been retained, recent expert proposals advocate moving toward the same neuroendocrine tumor framework [14].

Given that the application of the WHO 2010 neuroendocrine neoplasm definitions to population-based data appears highly heterogeneous, a scoping review is necessary to identify where conceptual drift occurs between clinical guidelines and registry-based research. Further, as a result of the rapid increase in database-driven publications and the evolving nature of neuroendocrine neoplasm nomenclature, a scoping approach is uniquely suited to provide a comprehensive overview of current reporting practices and to identify specific areas where terminology requires standardization.

### Aim

The aim of this scoping review was to map the utilization of neuroendocrine neoplasm nomenclature within research articles published from 2012 to 2022 which utilized SEER and/or the NCDB. Specifically, we sought to characterize the nature and extent of nomenclature discordance with the 2010 WHO classification in studies focusing on gastroenteropancreatic neuroendocrine tumors. The starting date of 2012 was chosen to give researchers 2 years to assimilate the updated classification system. The end date of 2022 was selected to capture a complete 10-year study period and directly aligns with the execution date of our comprehensive literature search. It is important to note that this scoping review focused exclusively on neuroendocrine tumors. Research articles limited to neuroendocrine carcinomas were excluded.

## 2. Methods

### 2.1. Study Design

This study was conducted as a scoping review to map the landscape of nomenclature usage in gastroenteropancreatic neuroendocrine tumor research. We followed the methodological framework for scoping reviews. This scoping review was designed and reported in accordance with the Preferred Reporting Items for Systematic Reviews and Meta-Analyses extension for Scoping Reviews (PRISMA-ScR) [15]; a completed PRISMA-ScR checklist is provided in Supplementary File S1. A formal review protocol was not registered, as scoping reviews are not eligible for registration in the PROSPERO international prospective register of systematic reviews; nonetheless, the review questions, eligibility criteria, and data-charting framework were defined a priori by the study team. In accordance with scoping review methodology, a formal critical appraisal or risk of bias assessment of the included studies was not performed, as the primary objective was to characterize reporting practices rather than synthesize clinical outcomes.

### 2.2. Inclusion and Exclusion Criteria

To be included in this review, articles had to be primary research published between 2012 and 2022. Studies were required to utilize data from the Surveillance, Epidemiology and End Results (SEER) program and/or the National Cancer Database (NCDB) to research primary gastroenteropancreatic neuroendocrine tumors. The following types of articles were excluded:

Articles other than primary research (e.g., reviews, letters, commentaries, case reports).

Articles not published in English or abstract-only records.

Studies focused on non-neuroendocrine tumors (e.g., adenocarcinomas) or non-gastroenteropancreatic neuroendocrine tumors.

Articles including only neuroendocrine carcinomas with no neuroendocrine tumors.

### 2.3. Search Strategy and Study Selection

A medical librarian (LHY) searched the literature for records including the concepts of neuroendocrine neoplasms, Surveillance Epidemiology End Results (SEER), and The National Cancer Database (NCDB). The librarian created search strategies using a combination of keywords and controlled vocabulary in Embase.com 1947–, Ovid Medline 1946–, Scopus 1823–, Cochrane Central Register of Controlled Trials (CENTRAL), The Cochrane Database of Systematic Reviews (CDSR), and Clinicaltrials.gov 1997–. All search strategies were completed on 4 February 2022, with no added limits and a total of 1639 results found. Overall, 558 duplicate records were deleted after using the de-duplication processes described in “De-duplication of database search results for systematic reviews in EndNote,” [16] and an additional 2 duplicates were removed using Covidence (Veritas Health Innovation, Melbourne, Australia) [17], resulting in a total of 1079 citations included in the project library.

The remaining articles’ abstracts were independently screened for relevance by two authors (TH and MH) and conflicts resolved by a third author (CWH) using covidence.org. The full text of the remaining articles was assessed according to the inclusion and exclusion criteria. Fully reproducible search strategies for each database are provided in Supplementary File S2. Inter-reviewer agreement was high at both stages. Cohen’s κ was computed from the Covidence inter-rater reliability report on the records that both primary reviewers (TH and MH) independently screened, 1029 of the 1079 records at title/abstract and 388 of the 421 full texts; records that did not receive two independent votes from this pair (those screened by a single reviewer or adjudicated by the senior author) fall outside the κ denominator. On this set, the reviewers agreed on 994 of 1029 dually screened records (96.6%; Cohen’s κ = 0.93) and, at full text, on 368 of 388 (94.8%; κ = 0.89); the 35 and 20 disagreements, respectively, were resolved by adjudication with the senior author (CWH).

### 2.4. Data Charting

Data charting and the assignment of each study to the nomenclature categories were performed independently and in duplicate by two authors (TH and MH), with discrepancies resolved by consensus and adjudicated by the senior author (CWH) when needed. Because category assignment followed the explicit, criterion-based definitions below (registry morphology codes and the differentiation field), most studies classified unambiguously; studies with borderline or ambiguous coding or differentiation reporting were re-examined jointly against these criteria, and any that remained unresolved were adjudicated by the senior author. To map the landscape of neuroendocrine neoplasm nomenclature usage, a standardized charting form was used to record the following information from each article:

Study Characteristics: Author, year of publication, citation title, journal, database utilized (SEER or NCDB), and study period.

Nomenclature and Classification Variables: International Classification of Diseases (ICD) topographic and morphology numbers and whether neuroendocrine neoplasm-specific taxonomy was retrieved directly from the cancer registries.

Grading and Differentiation Data: The number of tumors recorded under specific differentiation grades (well, moderately, poorly, or undifferentiated) and whether the article referred to neuroendocrine tumors as “carcinoids”.

The primary goal of this charting process was to classify the literature into a descriptive framework consisting of four categories to assess the nature and extent of nomenclature discordance, with a secondary goal of capturing the persistence of “carcinoid” terminology in a fifth category:

1. Insufficient Data: Articles lacking the necessary tumor grade or ICD codes to assess nomenclature accuracy.
2. Appropriate Interpretation: Articles where terminology was used consistently with the 2010 WHO classification.
3. Inclusion of Poorly/Undifferentiated Neoplasms: Articles grouping poorly or undifferentiated neoplasms under the “neuroendocrine tumor” designation. Studies that reported neuroendocrine tumors and neuroendocrine carcinomas but analyzed them as separate cohorts were classified on the basis of the well-differentiated cohort and were not counted in this category.
4. Conflation of Grading Systems: Articles reporting neuroendocrine neoplasm-specific grades (G1, G2, G3) derived from database differentiation grades rather than proliferation markers.
5. Historical Terminology: Articles utilizing the outdated term “carcinoid” to describe neuroendocrine tumors.

The full extracted dataset is provided in Supplementary Tables S1–S5.

### 2.5. Forward Citation Analysis

To assess whether the nomenclature discordances identified in this review have been cited in the broader literature, we performed a forward citation analysis of the 124 unique discordant studies cataloged in Supplementary Tables S3 and S4.

Each study was resolved to a Digital Object Identifier (DOI) using Crossref and PubMed; matches with a title-similarity score below 0.90 or with Crossref/PubMed disagreement were verified manually by the senior author, and seven incorrect DOIs were corrected.

Semantic Scholar [18] provides an influentialCitationCount metric which algorithmically flags citations that substantively build upon the cited work rather than passingly mentioning it. This count was captured for each study.

Citing works were classified as reviews or meta-analyses if flagged as such by OpenAlex [19], by a PubMed publication type of “Review,” “Systematic Review,” or “Meta-Analysis,” or by review-specific terminology in the title. To assess citation by clinical guidance, we intersected the reference lists of 13 major neuroendocrine tumor guideline and consensus documents (National Comprehensive Cancer Network [NCCN], European Society for Medical Oncology [ESMO], European Neuroendocrine Tumor Society [ENETS], North American Neuroendocrine Tumor Society [NANETS], UK and Ireland Neuroendocrine Tumour Society [UKINETS], and WHO classifications) with the discordant-study set; guidelines with fewer than 30 entries in their OpenAlex reference list were hand-checked against the source manuscript. For NCCN-cited discordant studies, the citing-sentence text and parent section heading were manually verified against the current edition of the NCCN Neuroendocrine and Adrenal Tumors guideline (v1.2026) [20].

### 2.6. Statistical Analysis

Inter-reviewer agreement at the title/abstract and full-text screening stages was quantified with Cohen’s κ. The temporal trend in the proportion of assessable studies showing nomenclature discordance was assessed with the Cochran–Armitage test for trends across annual data from 2013 to 2021 (2022 excluded as a partial year). Citation counts are summarized as medians with interquartile ranges. Analyses were performed in Python 3.13 (NumPy 2.3.4, SciPy 1.16.2).

## 3. Results

### 3.1. Study Selection and Mapping Characteristics

**Figure 1:**
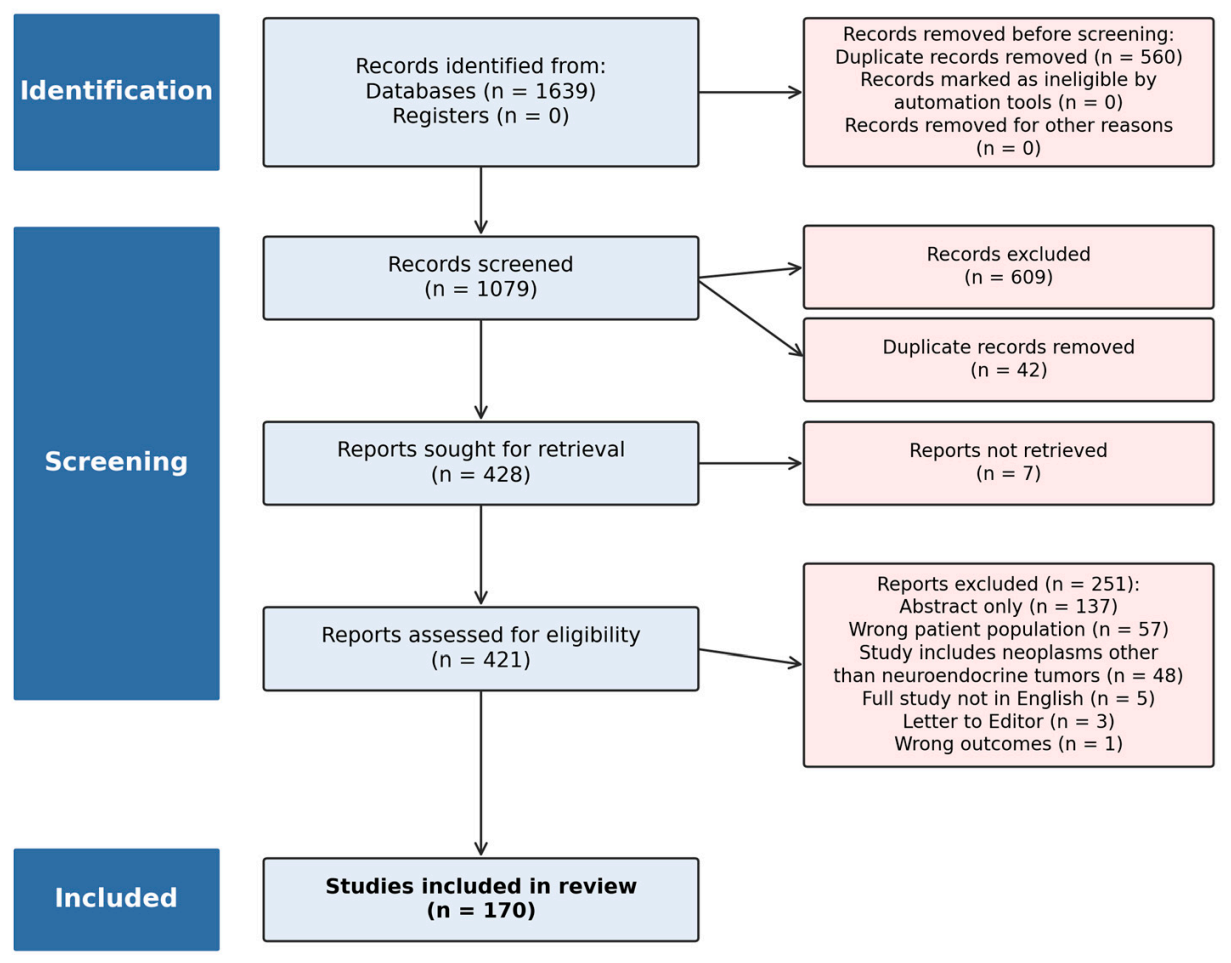
PRISMA-ScR flowchart of study selection and inclusion.

The initial search yielded 1639 results, refined to 1079 records after de-duplication. Of these, title and abstract screening excluded 609, a further 42 were flagged as duplicates, and 7 reports could not be retrieved, leaving 421 full-text articles assessed for eligibility. A total of 170 articles met all inclusion criteria and were included in this scoping review to map the utilization of neuroendocrine nomenclature. Figure 1 illustrates the PRISMA-ScR flow of study identification and selection. The majority of these studies utilized the SEER database (*n* = 123, 72.4%), while the remaining 47 studies (27.6%) utilized the NCDB. These studies represented a wide range of research focuses, with the majority of the mapped literature specifically examining pancreatic neuroendocrine tumors (*n* = 71, 41.8%). Other frequently studied sites included the small bowel, duodenum and ampulla (*n* = 21); colorectal (*n* = 19); and appendix (*n* = 10), while 38 studies focused on multiple gastroenteropancreatic sites simultaneously (Table 3).

### 3.2. Landscape of Nomenclature Usage

**Figure 2:**
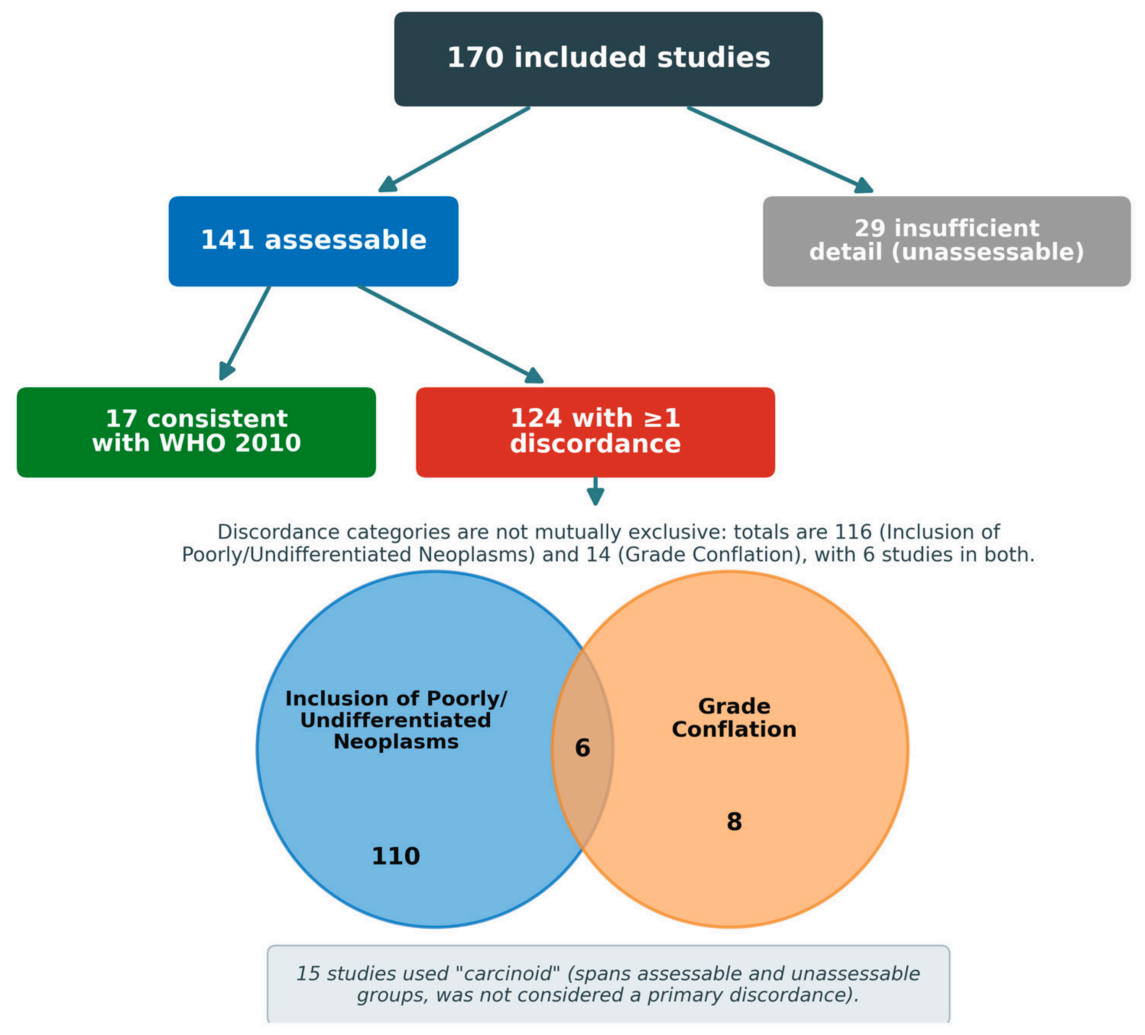
Classification of the 170 included studies by nomenclature concordance with the 2010 WHO framework.

The charting process revealed significant heterogeneity and widespread discordance in how the 2010 WHO nomenclature is applied within the published literature. Of the 170 included studies, 90% either demonstrated at least one instance of nomenclature discordance (*n* = 124) or provided insufficient methodological detail to confirm the appropriate use of terminology (*n* = 29, Supplementary Table S1). Among the remaining 141 studies that provided enough detail in the methods to judge nomenclature, only 12% (*n* = 17) utilized neuroendocrine nomenclature consistently with the 2010 WHO guidelines (Supplementary Table S2). The overall landscape of nomenclature usage across the literature is categorized in Figure 2. Within the 141 assessable studies, true nomenclature discordance was characterized by two primary errors.

### 3.3. Inclusion of Neuroendocrine Carcinomas

The most prevalent finding in the landscape was the inclusion of poorly differentiated and undifferentiated neoplasms (histologically defined as neuroendocrine carcinomas) in studies purportedly focused on neuroendocrine tumors. This was observed in 116 studies (82.3% of assessable papers). In the 107 that reported differentiation, an average of 18.7% of the included neoplasms were coded as poorly differentiated or undifferentiated, indicating the inclusion of more aggressive poorly or undifferentiated neoplasms within neuroendocrine tumor-specific cohorts (Supplementary Table S3).

### 3.4. Conflation of Grading Systems

A significant conceptual drift was identified regarding the term “grade.” Fourteen articles (9.9% of assessable papers) appeared to conflate database-specific differentiation grades (Grades 1–4) provided by SEER/NCDB with WHO proliferation-specific grades (G1, G2, and G3). These studies reported G1–G3 classifications despite utilizing database versions that lacked the mitotic count or Ki-67 index data required to make such distinctions (Supplementary Table S4). Notably, six articles demonstrated both the inclusion of poorly differentiated neoplasms and the conflation of grading systems.

These two errors involve the same registry differentiation field in opposite ways. The inclusion of poorly or undifferentiated neoplasms is a cohort-composition error, a failure to exclude poorly differentiated (grade 3) or undifferentiated (grade 4) cases on the differentiation field. Grade conflation, by contrast, is an interpretation error, in which that same differentiation field was treated as if it were the WHO proliferation grade. Inclusion was by far the more prevalent (116 vs. 14 studies).

### 3.5. Methodological Opacity and Historical Terminology

A substantial portion of the literature (17%, *n* = 29) did not include sufficient information in the methods section to assess the adequacy of nomenclature usage. Furthermore, while not categorized as a primary discordance with the 2010 framework, the outdated term “carcinoid” persisted in 8.8% (*n* = 15 of 170 total articles) of the articles reviewed (Supplementary Table S5). Notably, only one of the 15 articles using “carcinoid” [21] was otherwise consistent with the 2010 WHO nomenclature; the remaining 14 exhibited inclusion of poorly or undifferentiated neoplasms, grading system conflation, or insufficient methodological detail. Residual carcinoid terminology rarely appears in isolation from other forms of nomenclature imprecision.

### 3.6. Temporal Trends in Nomenclature Usage

**Figure 3:**
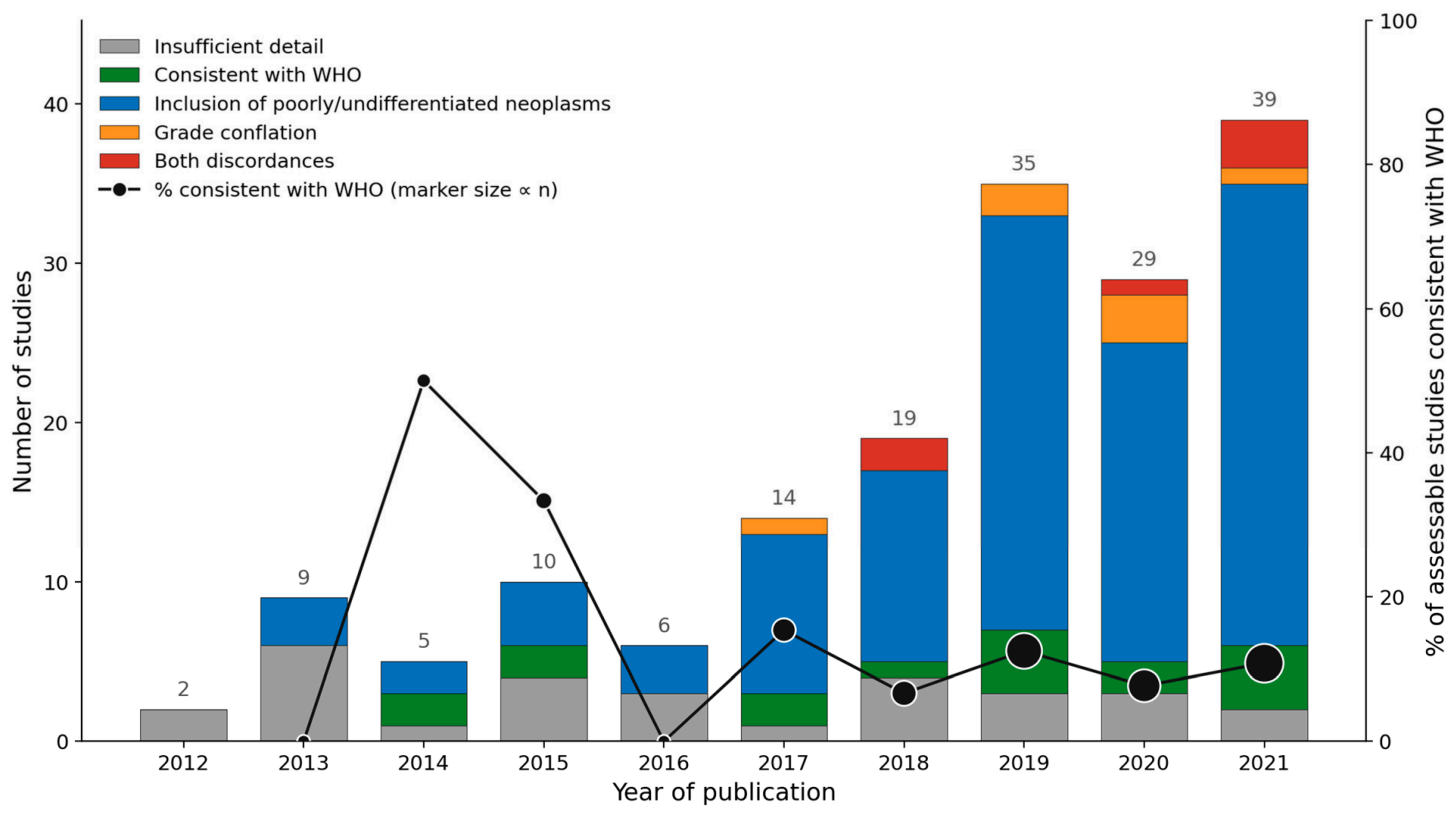
Number of studies in each category by year of publication. The overlaid line shows the percentage of each year’s assessable studies consistent with the 2010 WHO nomenclature; marker size is proportional to that year’s number of assessable studies. There was no significant temporal trend in discordance (Cochran–Armitage test, *p* = 0.14); 2022 was excluded as a partial year.

Mapping the data over time (2012–2021) shows that the volume of gastroenteropancreatic neuroendocrine tumor research using national databases increased substantially, while nomenclature accuracy did not improve (Figure 3). The literature is concentrated in recent years; more than 60% of all studies were published in 2019–2021. The earlier years contribute few assessable studies (none in 2012 and only three to six per year in 2013–2016), so their annual rates are unstable. We therefore focused on the years with an adequate annual sample. Across 2017–2021, 89.4% of assessable studies (110 of 123) contained at least one discordance, and the proportion consistent with the 2010 WHO nomenclature remained low throughout (7–15% per year). A Cochran–Armitage test across 2013–2021 found no significant temporal trend in discordance (*p* = 0.14), and the point estimate did not indicate improvement. Nomenclature discordance was therefore a stable feature of the literature across the 2012–2022 study period rather than a residue of the early post-2010 transition period.

### 3.7. Forward Citations of Discordant Studies

To assess downstream citation exposure, we performed a forward citation analysis on the 124 unique studies exhibiting at least one of the two principal discordances (Supplementary Tables S3 and S4). Across these 124 studies, a total of 7323 forward citations from 5145 unique citing works were identified.

**Figure 4:**
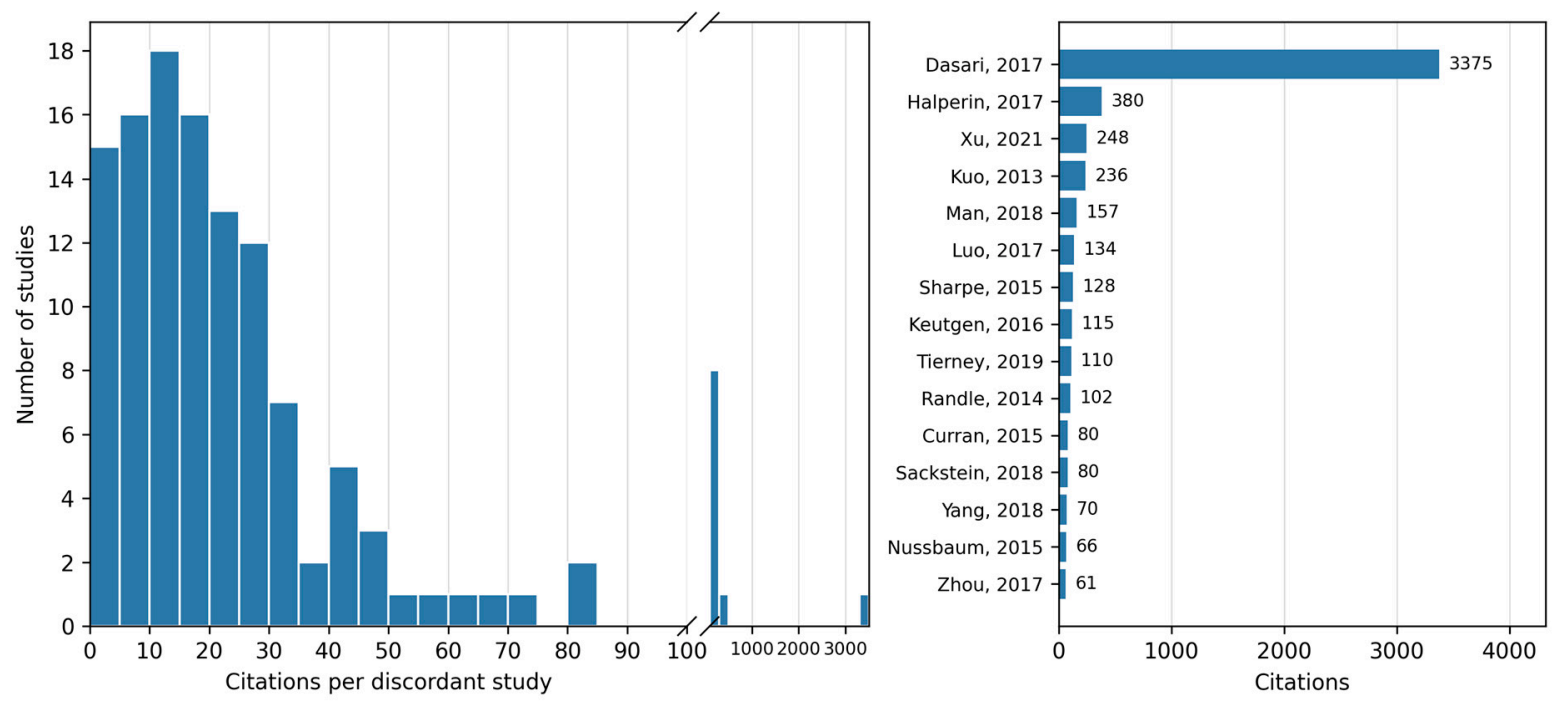
Downstream citation impact of discordant database studies. (**Left panel**) Distribution of forward citations across the 124 discordant studies, utilizing a broken *x*-axis to accommodate outliers. (**Right panel**) The 15 most-cited discordant studies, ranked by total OpenAlex citation count.

Citation impact within this set was heavily skewed by a single paper. A 2017 study on neuroendocrine tumor incidence and survival [22] accounted for 3375 citations, representing 46.1% of all forward citations in the discordant set (Figure 4). The median citation count across the 124 studies was 18 (interquartile range, 9 to 32), and 122 of the 124 studies (98.4%) had been cited at least once. Excluding this single outlier, the remaining 123 discordant studies still accounted for 3948 forward citations from 2790 unique citing works, indicating that downstream exposure does not rest on any one paper. These quantities are overlapping subsets of the same citing corpus, not additive: the 7323 citations are individual citation events arising from 5145 unique citing works, and the review, influential, and guideline citations described below are counted within this same corpus rather than in addition to it.

Discordant literature has frequently been incorporated into secondary research. Of the 124 studies, 109 (87.9%) were cited by at least one review or meta-analysis, encompassing 1075 distinct review articles. Furthermore, 64 of the 124 discordant studies (51.6%) received at least one citation flagged by Semantic Scholar’s influentialCitationCount metric as substantively building upon the cited work (460 such influential citations in total). An influential-citation flag indicates that the citing work drew substantively on the cited study, but it does not establish that the citing work relied specifically on the nomenclature-affected component.

Direct citation by clinical practice guidelines and consensus documents was also observed. Of the 13 major guideline and consensus documents queried, four directly cited a total of six unique discordant papers. These included the NCCN Neuroendocrine and Adrenal Tumors guideline (v2.2021) [23], which cited four discordant studies; the ESMO Clinical Practice Guidelines [24]; and two ENETS 2023 guidance papers for functioning [25] and nonfunctioning [26] pancreatic neuroendocrine tumors. The most-cited discordant study [22] appeared in three of the four implicated guideline documents. Direct review of the current NCCN Neuroendocrine and Adrenal Tumors guideline (v1.2026) confirmed that all four citations persist; three of the four [27,28,29] appear in the Staging section as prognostic and validation evidence for the American Joint Committee on Cancer (AJCC) TNM system, while only Dasari et al. [22] is confined to background framing.

## 4. Discussion

This scoping review of 170 studies published from 2012 to 2022 reveals discordance between international neuroendocrine neoplasm nomenclature guidelines and their application in research utilizing national cancer registries that was widespread and persistent across the study period. Despite the 2010 WHO update intended to standardize these definitions, 88% of 141 assessable studies failed to apply the nomenclature accurately. This suggests a significant “conceptual drift” where the limitations of database coding appear to have been systematically conflated with clinically meaningful classification.

### 4.1. Registry Choice and Systemic Discordance

Our mapping of the literature highlights a heavy reliance on the SEER database, which was used in over 72% of the included studies (*n* = 123). This is significant because SEER data prior to 2021 lacks the proliferation markers (mitotic count and Ki-67 index) necessary for accurate WHO grading [11]; these registry limitations for neuroendocrine neoplasm epidemiology have been noted previously [10]. Furthermore, the fact that nomenclature discordance was identified across all topographic sites, from the frequently studied pancreas (41.8%) to rarer sites such as the esophagus and gallbladder, suggests that the conceptual drift between registry codes and clinical guidelines is a systemic issue within the field, rather than one confined to a specific organ or database. The most concrete consequence of this discordance is the composition of the cohorts themselves: many registry-based “neuroendocrine tumor” studies include patients whose tumors would not meet WHO criteria for a neuroendocrine tumor.

These difficulties are not unique to neuroendocrine neoplasms: registry coding schemes lag or blur evolving pathological classifications across oncology, and database studies performed before the coding is reconciled inherit the mismatch. A prominent example is the 2016 reclassification of the noninvasive encapsulated follicular variant of papillary thyroid carcinoma as noninvasive follicular thyroid neoplasm with papillary-like nuclear features, which removed a large group of indolent tumors from the “carcinoma” category; registry-based analyses conducted before this change had overcounted thyroid carcinoma and contributed to overtreatment [30]. In the biliary tract, inconsistent definitions and ICD-O coding of intrahepatic, hilar, and Klatskin tumors have similarly confounded registry-based estimates of cholangiocarcinoma incidence [31]. The general lesson, applicable well beyond neuroendocrine neoplasms, is that registry codes must be verified against the current classification before they are used to define a study cohort.

### 4.2. Inclusion of Poorly or Undifferentiated Neoplasms in Neuroendocrine Tumor Research

The most critical finding in our map of the literature is the systematic inclusion of poorly differentiated and undifferentiated neoplasms within neuroendocrine tumor-specific cohorts. According to the 2010 definitions, these aggressive neoplasms are neuroendocrine carcinomas, which are biologically and clinically distinct from well-differentiated neuroendocrine tumors [32]. At the molecular level, neuroendocrine carcinomas are characteristically driven by TP53 and RB1 inactivation, whereas well-differentiated neuroendocrine tumors typically retain wild-type TP53 and RB1 and, in pancreatic primaries, often carry ATRX, DAXX, or MEN1 alterations [33]. With an average poorly or undifferentiated neoplasm inclusion rate of 18.7% across the 107 studies that reported differentiation, much of the published literature regarding gastroenteropancreatic neuroendocrine tumors is inadvertently weighted by the far more aggressive behavior of poorly or undifferentiated neoplasms.

### 4.3. Grade Conflation: Proliferation Versus Differentiation

A second major theme is the conflation of the registry differentiation grade with the WHO proliferation grade. SEER (prior to 2021) and the NCDB (prior to 2018) use a 1–4 scale that describes how closely tumor cells resemble normal tissue, that is, their differentiation [11,12], and many researchers have treated these values as interchangeable with WHO G1, G2, and G3. They are not: WHO grading is defined strictly by proliferation (mitotic count or Ki-67 index), data that do not exist in these earlier registry versions. This is not a divergence between the AJCC and WHO systems, which are concordant, since AJCC staging for well-differentiated neuroendocrine tumors incorporates the WHO proliferation grade. The problem lies instead within the registry differentiation field itself, whose levels do not correspond to WHO G1–G3 at all; researchers simply assign such a correspondence.

The consequence is specific. When a study stratifies its cohort by the registry differentiation grade and relabels the strata G1, G2, and G3, its highest-grade arm (registry grade 3, and often grade 4) consists of poorly or undifferentiated neoplasms, that is, neuroendocrine carcinomas; a comparison of outcomes “by grade” is then actually a comparison of neuroendocrine tumors against neuroendocrine carcinomas, presented as an effect of proliferation grade. Where inclusion of poorly or undifferentiated neoplasms biases the data, grade conflation biases the interpretation, dressing a differentiation-based contrast as a proliferation-grade finding and reporting grades that were never measured. Differentiation and proliferation are, moreover, independent axes: a well-differentiated tumor may itself be highly proliferative (a Ki-67 above 20%, recognized in the 2022 WHO classification [8] as a well-differentiated neuroendocrine tumor G3). Such a tumor is still biologically a neuroendocrine tumor, molecularly distinct from a neuroendocrine carcinoma, yet it carries the same proliferation grade as a poorly differentiated carcinoma; proliferation grade alone does not separate them, and the registry differentiation field, which records only differentiation, can neither identify it nor stand in for the WHO grade.

### 4.4. Downstream Citation Exposure of Discordant Studies

The downstream citation analysis demonstrates that the nomenclature discordances identified in this review are not confined to isolated primary studies. With 87.9% of the discordant database studies cited by subsequent reviews and meta-analyses, discordant studies have received substantial citation exposure within the broader oncology literature; whether this exposure has reproduced the underlying nomenclature error in citing works is not established by citation counts alone. This exposure is heavily skewed by a small number of highly visible publications; a single 2017 study [22] accounted for 46.1% of all downstream citations. Crucially, 32.6% of the neoplasms included in this highly cited study were poorly or undifferentiated neoplasms, neuroendocrine carcinomas by WHO definition. However, even with this extreme outlier excluded, the remaining discordant studies still generated 3948 citations, indicating that this downstream citation exposure is broad rather than confined to a single paper. Because such heavily cited discordant studies have been incorporated into clinical practice guidelines and consensus documents, including NCCN, ESMO, and ENETS, there is a concrete mechanism by which registry-based nomenclature errors can skew the perceived aggressiveness of neuroendocrine tumors. Whether this citation exposure has influenced treatment recommendations is not established by these data; in the one guideline we examined in detail, the discordant studies were cited for staging validation and background rather than for therapy.

### 4.5. Potential Risk of Over-Treatment

These nomenclature errors are not merely semantic; they have the potential to bias clinical decision-making. In 2016, Tang et al. [32] demonstrated a disease-specific survival of 55 months in patients with well-differentiated gastroenteropancreatic neuroendocrine tumors with a high-grade component, compared with only 11 months in patients with poorly differentiated neuroendocrine carcinoma. Even in patients with advanced disease, the prognostic difference between neuroendocrine tumors and carcinomas is substantial. The two are also managed differently: well-differentiated neuroendocrine tumors are treated with somatostatin analogs, everolimus, or peptide receptor radionuclide therapy, whereas neuroendocrine carcinomas receive platinum-based chemotherapy, so misclassification can translate directly into inappropriate treatment [20,24]. When a registry-derived “neuroendocrine tumor” cohort includes a mean of 18.7% poorly or undifferentiated neoplasms, the resulting outcomes data are likely to overestimate the adverse prognosis for true neuroendocrine tumor patients. In principle, this amplified perception of risk could bias clinicians toward more aggressive treatment for patients with relatively indolent, well-differentiated disease. We emphasize, however, that this scoping review measured nomenclature usage and citation exposure only; whether these errors have actually altered treatment recommendations or produced over-treatment was not assessed and remains a hypothesis for future study.

### 4.6. Persistence of “Carcinoid”

The continued use of “carcinoid” in 8.8% of the literature, decades after the WHO discouraged it, marks the slow assimilation of standardized terminology. Beyond imprecision, the term carries misleading connotations when it is applied generically to gastroenteropancreatic neuroendocrine tumors [13]. Because “carcinoid” is bound to carcinoid syndrome, the label implies hormonal activity, yet most gastroenteropancreatic neuroendocrine tumors are non-functional [24]; a “carcinoid” designation can therefore lead clinicians to presume a functional tumor and to pursue unnecessary biochemical testing or pre-operative somatostatin prophylaxis against carcinoid crisis. We did not measure these downstream clinical effects and present them as illustrative rather than established. This does not mean that the term is always inappropriate: it remains the current designation for pulmonary typical and atypical carcinoid, although a shift toward neuroendocrine tumor terminology has been proposed even there [14], and for carcinoid syndrome itself. Precisely because the term retains these specific, legitimate meanings, its generic use for gastroenteropancreatic tumors imports them where they do not belong. For this review, “carcinoid” usage was recorded separately as a descriptive category rather than as one of the two primary discordances; consistent with its being a marker rather than a driver, 14 of the 15 studies using “carcinoid” also exhibited another form of nomenclature discordance or lacked sufficient detail to assess. Residual “carcinoid” usage therefore rarely occurred in isolation, tending to co-occur with, rather than independently produce, the more substantive errors.

### 4.7. A Reference Approach for Registry-Based Neuroendocrine Tumor Studies

The studies we classified as consistent with the 2010 WHO nomenclature (Supplementary Table S2) share several practices that can serve as a practical benchmark for future registry-based neuroendocrine tumor research. First, they restricted morphology (ICD-O-3) selection to well-differentiated neuroendocrine tumor codes and explicitly excluded poorly differentiated, small-cell, and large-cell neuroendocrine carcinoma codes, or analyzed any carcinomas as a separate cohort. Morphology-code selection alone is not sufficient, however: in practice, cohorts retrieved with the correct well-differentiated codes can still span the full range of registry differentiation grades (1 through 4), because that field is recorded independently of the morphology code. A rigorous cohort therefore also filters the differentiation field, retaining well-differentiated cases and excluding those recorded as poorly differentiated (grade 3) or undifferentiated (grade 4), which are by definition neuroendocrine carcinomas; moderately differentiated cases (grade 2) are intermediate, and either their inclusion or their exclusion is defensible so long as the choice is stated. Used in this way, for differentiation rather than as a proxy for proliferation, the differentiation field is informative; the error lies not in using it but in mistaking it for the WHO proliferation grade (Section 4.3). Second, none of these studies used the registry versions that carry proliferation information (NCDB from 2018; SEER from 2021); instead, they refrained from reporting proliferation-based WHO grades (G1–G3) when the underlying Ki-67 or mitotic data were unavailable, which was the appropriate choice for the data they had. As those newer versions mature, investigators will increasingly be able to assign WHO grade directly, although only for diagnosis years from the update onward (Section 3.6). Third, they reported the differentiation composition of the final cohort, including how intermediate-grade cases were handled, so that its makeup is transparent to readers. Table 4 summarizes the ICD-O-3 morphology codes most relevant to gastroenteropancreatic neuroendocrine neoplasms, and how they should be used to isolate neuroendocrine tumors, as a quick reference for investigators querying these registries.

### 4.8. Limitations of the Scoping Review

As with all scoping reviews, this study is limited by search-strategy bias: only studies indexed in the queried databases, returned by our specific search parameters, and published in English-language journals were eligible for inclusion. Furthermore, 17% of the identified literature provided such poor methodological detail that their nomenclature usage could not be assessed, which may indicate that the true rate of discordance is even higher than reported here. Finally, registry differentiation is assigned by the reporting pathologist rather than by central re-review, and the differentiation fields may be incomplete, historically variable, or subject to coding error; a poorly or undifferentiated designation nonetheless establishes that a neoplasm is not a well-differentiated neuroendocrine tumor, although we could not independently verify these pathological assessments.

Our literature search closed in February 2022, and it is possible that the discordance has resolved since. However, the registry updates are prospective: the NCDB (2018) and SEER (2021) proliferation fields populate only for diagnosis years from those updates onward, whereas the studies we identified pooled a median of 14 diagnosis years (range 5–43), with 91% spanning at least a decade. Therefore, until the updated coding has been in place for at least ten years (approximately 2028 for the NCDB and 2031 for SEER), at least a portion of every such cohort will likely still depend on the older differentiation coding, which must still be interpreted correctly. Accordingly, we have confined our claims regarding the prevalence and persistence of discordance to the 2012–2022 study period; we cannot directly characterize studies published after our search, and the structural argument above indicates only that the underlying source of discordance is unlikely to have resolved, not that its rate has necessarily remained unchanged.

Our forward citation analysis is limited by the inherent coverage variations among citation databases. OpenAlex counts were cross-validated against Semantic Scholar, with Google Scholar arbitration of divergent records, to establish a conservative lower bound of downstream exposure. Even so, certain non-journal artifacts such as the AJCC Cancer Staging Manual lack a digitally indexed reference list. Consequently, the true rate of citation by clinical guidelines may be higher than reported. Furthermore, capturing a downstream citation establishes exposure to the original nomenclature error but does not confirm whether the error was accepted uncritically by the citing authors.

### 4.9. Future Directions

Several operational steps follow from these findings. The most direct is that journals and guideline panels could require a brief nomenclature-compliance statement for registry-based submissions, confirming that cohort composition aligns with current WHO definitions. Beyond this, engagement with SEER and NCDB leadership could help refine grade-field instructions and surface the neuroendocrine tumor–carcinoma boundary at the query stage, and targeted educational efforts (society webinars and methods primers) could raise awareness among investigators who use these registries. Finally, a dedicated study contacting the corresponding authors of discordant studies to ascertain the rationale for their coding choices, together with a citation-context analysis of how discordant studies are actually used by citing works, would test whether the citation exposure documented here translates into propagated conceptual error. Such root-cause and citation-content investigations are distinct lines of inquiry beyond the scope of the present descriptive map. In particular, a prospective update of this review, once the post-2018 (NCDB) and post-2021 (SEER) coded literature has matured, would directly test whether the registry coding updates have improved nomenclature accuracy, an assessment that the 2012–2022 window cannot yet support.

## 5. Conclusions

Nomenclature discordance in registry-based gastroenteropancreatic neuroendocrine tumor research published between 2012 and 2022 was widespread, persistent throughout the study period, and present in studies cited by the secondary and guideline literature. Because the affected cohorts incorporate more aggressive poorly or undifferentiated neoplasms, their outcome estimates are likely to overstate the aggressiveness of well-differentiated neuroendocrine tumors, particularly for prognostic estimates, although this review did not quantify the magnitude of that effect on any individual study. Database studies are essential for understanding rare diseases such as gastroenteropancreatic neuroendocrine tumors, but they are only as good as the terminology used to query them. This review highlights a critical need for researchers to verify explicitly that cohort composition aligns with current WHO definitions rather than relying solely on registry-assigned histologic codes. Given the high rate of discordance, journals should consider requiring a nomenclature-compliance statement or checklist for studies utilizing national cancer registries to ensure results are aligned with current WHO definitions.

## Figures and Tables

**Table 1:** The 2010 WHO classification framework for gastroenteropancreatic neuroendocrine neoplasms (adapted from [6]).

	Differentiation	Designation	Grade	Mitotic Count (/10 HPF)	Ki 67 Index (%)
Neuroendocrine neoplasms	Well	*Neuroendocrine Tumor*	G1	<2	≤2
		*Neuroendocrine Tumor*	G2	2–20	3–20
	Poor	*Neuroendocrine Carcinoma* Large-cell Small-cell	G3	>20	>20

**Table 2:** The 2022 WHO classification framework for gastroenteropancreatic neuroendocrine neoplasms (adapted from [8]).

	Differentiation	Designation	Grade	Mitotic Count (/10 HPF)	Ki 67 Index (%)
Neuroendocrine neoplasms	Well	*Neuroendocrine Tumor*	G1	<2	<3
		*Neuroendocrine Tumor*	G2	2–20	3–20
		*Neuroendocrine Tumor*	G3	>20	>20
	Poor	*Neuroendocrine Carcinoma* Large-cell Small-cell		>20	>20

**Table 3:** Anatomic landscape of the mapped literature.

Anatomic Site	Number of Studies (%)
Pancreas	71 (41.8)
Multiple Sites	38 (22.4)
Small Bowel/Duodenum/Ampulla	21 (12.4)
Small Bowel	16 (9.4)
Duodenum	4 (2.4)
Ampulla	1 (0.6)
Colorectal	19 (11.2)
Rectal	11 (6.5)
Colon	3 (1.8)
Combined	5 (2.9)
Appendix	10 (5.9)
Gastric/Esophageal	8 (4.7)
Liver/Gallbladder	3 (1.8)

**Table 4:** Representative ICD-O-3 morphology codes relevant to case selection in registry-based neuroendocrine tumor studies. Codes are illustrative of the categories investigators should distinguish and should be verified against the current SEER/NCDB coding manuals.

Group	Representative ICD-O-3 Morphology Codes	Handling in a Neuroendocrine Tumor Cohort
Well-differentiated neuroendocrine tumor	8240/3, 8241/3, 8242/3, 8249/3 8150/3–8153/3, 8155/3–8156/3 (pancreatic functioning/islet-cell)	Include; filter on the registry differentiation field
Neuroendocrine carcinoma (poorly differentiated)	8246/3 (neuroendocrine carcinoma) 8041/3 (small-cell) 8013/3 (large-cell neuroendocrine carcinoma) 8045/3 (combined small-cell)	Exclude, or analyze separately
Mixed/neuroendocrine-differentiated	8244/3 (mixed neuroendocrine–non-neuroendocrine) 8154/3 (mixed pancreatic endocrine and exocrine tumor, malignant) 8574/3 (adenocarcinoma with neuroendocrine differentiation)	Not a well-differentiated neuroendocrine tumor; handle explicitly

## Data Availability

The data analyzed in this scoping review were extracted from previously published articles, all of which are cited in the manuscript. The full list of included studies, the data-charting form, and the complete database search strategies are provided as Supplementary Materials. Further derived data are available from the corresponding author upon reasonable request.

## Supplementary Materials

The following supporting information can be downloaded at https://www.mdpi.com/article/10.3390/cancers18152508/s1. File S1: PRISMA-ScR Checklist. File S2: Reproducible database search strategies for each database queried, together with the following data tables: Table S1, Articles with insufficient information to assess nomenclature use; Table S2, Articles consistent with WHO nomenclature; Table S3, Articles that include poorly and undifferentiated neoplasms as neuroendocrine tumors; Table S4, Articles reporting neuroendocrine-specific grades; Table S5, Articles that use the term “carcinoid”.

## References

[1] Oronsky B., Ma P.C., Morgensztern D., Carter C.A. (2017). Nothing but NET: A review of neuroendocrine tumors and carcinomas. Neoplasia.

[2] Creutzfeldt W. (1996). Carcinoid tumors: Development of our knowledge. World J. Surg..

[3] Klöppel G. (2017). Neuroendocrine neoplasms: Dichotomy, origin and classifications. Visc. Med..

[4] Jögi A., Vaapil M., Johansson M., Påhlman S. (2012). Cancer cell differentiation heterogeneity and aggressive behavior in solid tumors. Upsala J. Med. Sci..

[5] Cree I.A., Tan P.H., Travis W.D., Wesseling P., Yagi Y., White V.A., Lokuhetty D., Scolyer R.A. (2021). Counting mitoses: SI(ze) matters!. Mod. Pathol..

[6] Klöppel G. (2011). Classification and pathology of gastroenteropancreatic neuroendocrine neoplasms. Endocr. Relat. Cancer.

[7] Rindi G., Klimstra D.S., Abedi-Ardekani B., Asa S.L., Bosman F.T., Brambilla E., Busam K.J., de Krijger R.R., Dietel M., El-Naggar A.K. (2018). A common classification framework for neuroendocrine neoplasms: An International Agency for Research on Cancer (IARC) and World Health Organization (WHO) expert consensus proposal. Mod. Pathol..

[8] Rindi G., Mete O., Uccella S., Basturk O., La Rosa S., Brosens L.A.A., Ezzat S., de Herder W.W., Klimstra D.S., Papotti M. (2022). Overview of the 2022 WHO Classification of Neuroendocrine Neoplasms. Endocr. Pathol..

[9] Bosman F.T., Carneiro F., Hruban R.H., Theise N.D. (2010). WHO Classification of Tumours of the Digestive System.

[10] Panzuto F., Partelli S., Campana D., de Braud F., Spada F., Cives M., Tafuto S., Bertuzzi A., Gelsomino F., Bergamo F. (2024). Epidemiology of gastroenteropancreatic neuroendocrine neoplasms: A review and protocol presentation for bridging tumor registry data with the Italian Association for Neuroendocrine Tumors (Itanet) national database. Endocrine.

[11] National Cancer Institute (2021). Site-Specific Data Item: Ki-67 Index for NET of the Digestive System. Surveillance, Epidemiology, and End Results Program Coding and Staging Manual.

[12] American College of Surgeons (2018). National Cancer Database Participant User File Data Dictionary.

[13] Chetty R. (2008). Requiem for the term ‘carcinoid tumour’ in the gastrointestinal tract?. Can. J. Gastroenterol..

[14] Beasley M.B., Yatabe Y., Papotti M., Cooper W.A., Derks J.L., Fernandez-Cuesta L., Kasajima A., Lantuejoul S., Pelosi G., Rekhtman N. (2026). IASLC Update on Classification of Pulmonary Neuroendocrine Neoplasms. J. Thorac. Oncol..

[15] Tricco A.C., Lillie E., Zarin W., O’Brien K.K., Colquhoun H., Levac D., Moher D., Peters M.D.J., Horsley T., Weeks L. (2018). PRISMA Extension for Scoping Reviews (PRISMA-ScR): Checklist and Explanation. Ann. Intern. Med..

[16] Bramer W.M., Giustini D., de Jonge G.B., Holland L., Bekhuis T. (2016). De-duplication of database search results for systematic reviews in EndNote. J. Med. Libr. Assoc..

[17] Veritas Health Innovation (2022). Covidence Systematic Review Software.

[18] Kinney R., Anastasiades C., Authur R., Beltagy I., Bragg J., Buraczynski A., Cachola I., Candra S., Chandrasekhar Y., Cohan A. (2023). The Semantic Scholar Open Data Platform. arXiv.

[19] Priem J., Piwowar H., Orr R. (2022). OpenAlex: A Fully-Open Index of Scholarly Works, Authors, Venues, Institutions, and Concepts. arXiv.

[20] National Comprehensive Cancer Network (2026). NCCN Clinical Practice Guidelines in Oncology: Neuroendocrine and Adrenal Tumors, Version 1.2026.

[21] Kachare S.D., Liner K.R., Vohra N.A., Zervos E.E., Fitzgerald T.L. (2014). A modified duodenal neuroendocrine tumor staging schema better defines the risk of lymph node metastasis and disease-free survival. Am. Surg..

[22] Dasari A., Shen C., Halperin D., Zhao B., Zhou S., Xu Y., Shih T., Yao J.C. (2017). Trends in the incidence, prevalence, and survival outcomes in patients with neuroendocrine tumors in the United States. JAMA Oncol..

[23] Shah M.H., Goldner W.S., Benson A.B., Bergsland E., Blaszkowsky L.S., Brock P., Chan J., Das S., Dickson P.V., Fanta P. (2021). Neuroendocrine and Adrenal Tumors, Version 2.2021, NCCN Clinical Practice Guidelines in Oncology. J. Natl. Compr. Cancer Netw..

[24] Pavel M., Öberg K., Falconi M., Krenning E., Sundin A., Perren A., Berruti A. (2020). Gastroenteropancreatic neuroendocrine neoplasms: ESMO Clinical Practice Guidelines for diagnosis, treatment and follow-up. Ann. Oncol..

[25] Hofland J., Falconi M., Christ E., Castaño J.P., Faggiano A., Lamarca A., Perren A., Petrucci S., Prasad V., Ruszniewski P. (2023). European Neuroendocrine Tumor Society 2023 guidance paper for functioning pancreatic neuroendocrine tumour syndromes. J. Neuroendocrinol..

[26] Kos-Kudła B., Castaño J.P., Denecke T., Grande E., Kjaer A., Koumarianou A., de Mestier L., Partelli S., Perren A., Stättner S. (2023). European Neuroendocrine Tumour Society (ENETS) 2023 guidance paper for nonfunctioning pancreatic neuroendocrine tumours. J. Neuroendocrinol..

[27] Chagpar R., Chiang Y.J., Xing Y., Cormier J.N., Feig B.W., Rashid A., Chang G.J., You Y.N. (2013). Neuroendocrine tumors of the colon and rectum: Prognostic relevance and comparative performance of current staging systems. Ann. Surg. Oncol..

[28] Curran T., Pockaj B.A., Gray R.J., Halfdanarson T.R., Wasif N. (2015). Importance of lymph node involvement in pancreatic neuroendocrine tumors: Impact on survival and implications for surgical resection. J. Gastrointest. Surg..

[29] Qadan M., Ma Y., Visser B.C., Kunz P.L., Fisher G.A., Norton J.A., Poultsides G.A. (2014). Reassessment of the current American Joint Committee on Cancer staging system for pancreatic neuroendocrine tumors. J. Am. Coll. Surg..

[30] Nikiforov Y.E., Seethala R.R., Tallini G., Baloch Z.W., Basolo F., Thompson L.D.R., Barletta J.A., Wenig B.M., Al Ghuzlan A., Kakudo K. (2016). Nomenclature Revision for Encapsulated Follicular Variant of Papillary Thyroid Carcinoma: A Paradigm Shift to Reduce Overtreatment of Indolent Tumors. JAMA Oncol..

[31] Welzel T.M., McGlynn K.A., Hsing A.W., O’Brien T.R., Pfeiffer R.M. (2006). Impact of classification of hilar cholangiocarcinomas (Klatskin tumors) on the incidence of intra- and extrahepatic cholangiocarcinoma in the United States. J. Natl. Cancer Inst..

[32] Tang L.H., Untch B.R., Reidy D.L., O’Reilly E., Dhall D., Jih L., Basturk O., Allen P.J., Klimstra D.S. (2016). Well-differentiated neuroendocrine tumors with a morphologically apparent high-grade component: A pathway distinct from poorly differentiated neuroendocrine carcinomas. Clin. Cancer Res..

[33] Mafficini A., Scarpa A. (2019). Genetics and epigenetics of gastroenteropancreatic neuroendocrine neoplasms. Endocr. Rev..

