# Supplementary material for: Mapping Misconceptions in Neuroendocrine Tumor Nomenclature: A Scoping Review of National Database Studies and Clinical Implications
Source: Cancers (Basel). 2026 Aug 5;18(15):2508. doi: 10.3390/cancers18152508 (PMC13464803; doi:10.3390/cancers18152508)
Supplement: Supplementary file 1 [file cancers-18-02508-s001.zip › Supplementary File S1.pdf]

Supplementary Material File S1.PRISMA-ScR Checklist

Reporting follows the PRISMA Extension for Scoping Reviews (PRISMA-ScR; Tricco et al., Ann Intern Med. 2018;169:467–473). Items 12 and 16 (critical appraisal) are optional and were not performed, consistent with scoping-review methodology.

Section	Item	PRISMA-ScR Checklist Item	Reported location
**Title**	1	Identify the report as a scoping review.	Title — identifies the work as "A Scoping Review of National Database Studies and Clinical Implications."
**Abstract**	2	Provide a structured summary that includes (as applicable): background, objectives, eligibility criteria, sources of evidence, charting methods, results, and conclusions that relate to the review questions and objectives.	Abstract; Simple Summary.
**Introduction**	3	Describe the rationale for the review in the context of what is already known. Explain why the review questions/objectives lend themselves to a scoping review approach.	Introduction — closing paragraphs justify a scoping-review approach given heterogeneous application of the 2010 WHO definitions.
	4	Provide an explicit statement of the questions and objectives being addressed with reference to their key elements (e.g., population or participants, concepts, and context) or other relevant key elements used to conceptualize the review questions and/or objectives.	Aim — population (GEP-NET registry studies), concept (concordance with 2010 WHO nomenclature), context (SEER and/or NCDB, 2012–2022).
**Methods**	5	Indicate whether a review protocol exists; state if and where it can be accessed (e.g., a Web address); and if available, provide registration information, including the registration number.	Methods, Study design — protocol and registration statement.
	6	Specify characteristics of the sources of evidence used as eligibility criteria (e.g., years considered, language, and publication status), and provide a rationale.	Methods, Inclusion and exclusion criteria — primary research, 2012–2022, English, GEP-NET, SEER/NCDB; exclusions enumerated.
	7	Describe all information sources in the search (e.g., databases with dates of coverage and contact with authors to identify additional sources), as well as the date the most recent search was executed.	Methods, Search strategy and study selection — Embase, Ovid MEDLINE, Scopus, Cochrane CENTRAL, CDSR, ClinicalTrials.gov with coverage dates; search executed 4 February 2022.
	8	Present the full electronic search strategy for at least 1 database, including any limits used, such that it could be repeated.	Methods, Search strategy and study selection; fully reproducible strategies for every database provided in the Supplementary Materials.
	9	State the process for selecting sources of evidence (i.e., screening and eligibility) included in the scoping review.	Methods, Search strategy and study selection — dual independent abstract screening (TH, MH); conflicts resolved by a third author (CWH) in Covidence; full text assessed against criteria.
	10	Describe the methods of charting data from the included sources of evidence (e.g., calibrated forms or forms that have been tested by the team before their use, and whether data charting was done independently or in duplicate) and any processes for obtaining and confirming data from investigators.	Methods, Data charting — standardized charting form applied independently in duplicate (TH, MH); discrepancies resolved by consensus.

Section	Item	PRISMA-ScR Checklist Item	Reported location
	11	List and define all variables for which data were sought and any assumptions and simplifications made.	Methods, Data charting — study characteristics; ICD topography/morphology codes and registry taxonomy; grading/differentiation data; “carcinoid” usage; the five charting categories are defined.
	12	[OPTIONAL] If done, provide a rationale for conducting a critical appraisal of included sources of evidence; describe the methods used and how this information was used in any data synthesis (if appropriate).	Not applicable — consistent with scoping-review methodology, no critical appraisal or risk-of-bias assessment was performed (stated in Methods, Study design).
	13	Describe the methods of handling and summarizing the data that were charted.	Methods, Data charting (descriptive categorical framework) and Forward citation analysis (DOI resolution via Crossref/PubMed; Semantic Scholar influential-citation metric; OpenAlex review classification; intersection with guideline reference lists).
**Results**	14	Give numbers of sources of evidence screened, assessed for eligibility, and included in the review, with reasons for exclusions at each stage, ideally using a flow diagram.	Results, Study selection and mapping characteristics — 1,639 → 1,079 de-duplicated → 421 full-text → 170 included; Figure 1 (PRISMA-ScR flow diagram).
	15	For each source of evidence, present characteristics for which data were charted and provide the citations.	Results (database split: SEER 72.4%, NCDB 27.6%) and Table 3 (anatomic distribution); per-study characteristics and citations in Supplementary Tables S1–S5.
	16	[OPTIONAL] If done, present data on critical appraisal of included sources of evidence (see item 12).	Not applicable — no critical appraisal was performed (see item 12).
	17	For each included source of evidence, present the relevant data that were charted that relate to the review questions and objectives.	Per-study charted data (nomenclature category, misclassification %, “carcinoid” use, forward-citation counts) in Supplementary Tables S1–S5.
	18	Summarize and/or present the charting results as they relate to the review questions and objectives.	Results — Landscape of nomenclature usage, inclusion of neuroendocrine carcinomas (82.3%), conflation of grading systems (9.9%), temporal trends, and forward citation analysis; Figures 2–5.
**Discussion**	19	Summarize the main results (including an overview of concepts, themes, and types of evidence available), link to the review questions and objectives, and consider the relevance to key groups.	Discussion — opening synthesis and themed subsections; relevance to researchers, guideline panels, and clinicians.
	20	Discuss the limitations of the scoping review process.	Discussion, Limitations of the Scoping Review.
	21	Provide a general interpretation of the results with respect to the review questions and objectives, as well as potential implications and/or next steps.	Conclusion — interpretation plus the recommended nomenclature-compliance statement/checklist for registry-based studies.
**Funding**	22	Describe sources of funding for the included sources of evidence, as well as sources of funding for the scoping review. Describe the role of the funders of the scoping review.	Back matter, Funding — “This research received no external funding”; funding of the individual included studies was not separately assessed (not applicable).
