# Supplementary material for: Mapping Misconceptions in Neuroendocrine Tumor Nomenclature: A Scoping Review of National Database Studies and Clinical Implications
Source: Cancers (Basel). 2026 Aug 5;18(15):2508. doi: 10.3390/cancers18152508 (PMC13464803; doi:10.3390/cancers18152508)
Supplement: Supplementary file 1 [file cancers-18-02508-s001.zip › Supplementary File S2 - Search Strategies and Data Tables.pdf]

Supplementary Materials

Mapping misconceptions in neuroendocrine tumor nomenclature: A scoping review of national database studies and clinical implications.

Hanson T, Hua M, Zarate Rodriguez J, Yaeger L, Chilukuri S, Trikalinos NA, Hammill C.

Supplementary Methods. Reproducible database search strategies.

The literature search was designed and executed by a medical librarian (Lauren H. Yaeger, MA, MLIS; Bernard Becker Medical Library, Washington University School of Medicine in St. Louis) on February 4, 2022. The full reproducible search strings used for each database queried are provided below.

Full Search Strategies

<u>Embase</u>
Date Searched: 2/4/2022

Applied Database Supplied Limits: 2012-2022

Number of Results: 623

Full Search Strategy:

('neuroendocrine carcinoma'/exp OR 'neuroendocrine tumor'/exp OR ((neuroendocrine OR ‘neuro-endocrine’) NEAR/3 (carcinoma* OR tumor* OR tumour* OR neoplasm*)):ti,ab,kw) AND ('national cancer database'/exp OR ('national cancer database' OR NCDB):ti,ab,kw OR ('Surveillance Epidemiology' NEAR/1 'End Results'):ti,ab,kw OR (SEER NEAR/3 (data OR database OR program OR collection OR statistic* OR software)):ti,ab,kw) AND [2012-2022]/py

<u>Ovid Medline</u>
Date Searched: 2/4/2022
Applied Database Supplied Limits: limit to yr="2012 - 2022"
Number of Results: 569

Full Search Strategy:

(exp Carcinoma, Neuroendocrine/ OR exp Neuroendocrine Tumors/ OR ((neuroendocrine OR neuro-endocrine) ADJ3 (carcinoma* OR tumor* OR tumour* OR neoplasm*)).ti,ab,kf.) AND ((national cancer database OR NCDB).ti,ab,kf. OR (Surveillance Epidemiology ADJ1 End Results).ti,ab,kf. OR (SEER ADJ3 (data OR database OR program OR collection OR statistic* OR software)).ti,ab,kf.)

limit to yr="2012 - 2022"

Scopus
Date Searched: 2/4/2022
Applied Database Supplied Limits: 2012-2022
Number of Results: 422

Full Search Strategy:

((TITLE-ABS-KEY((neuroendocrine OR “neuro-endocrine”) W/3 (carcinoma* OR tumor* OR tumour* OR neoplasm*)))) AND ((TITLE-ABS-KEY(“national cancer database” OR NCDB)) OR (TITLE-ABS-KEY(“Surveillance Epidemiology” W/1 “End Results”)) OR (TITLE-ABS-KEY(SEER W/3 (data OR database OR program OR collection OR statistic* OR software)))) AND (LIMIT-TO (PUBYEAR , 2022) OR LIMIT-TO (PUBYEAR , 2021) OR LIMIT-TO (PUBYEAR , 2020) OR LIMIT-TO (PUBYEAR , 2019) OR LIMIT-TO (PUBYEAR , 2018) OR LIMIT-TO (PUBYEAR , 2017) OR LIMIT-TO (PUBYEAR , 2016) OR LIMIT-TO (PUBYEAR , 2015) OR LIMIT-TO (PUBYEAR , 2014) OR LIMIT-TO (PUBYEAR , 2013) OR LIMIT-TO (PUBYEAR , 2012))

The Cochrane Library

Date Searched: 2/4/2022
Applied Database Supplied Limits: Cochrane Library publication date from Jan 2012 to Feb 2022, Publication Year from 2012 to 2022, in Trials
Number of Results:

CENTRAL: 16

CDSR: 0

Full Search Strategy:

([mh "Carcinoma, Neuroendocrine"] OR [mh "Neuroendocrine Tumors"] OR ((neuroendocrine OR "neuro endocrine") NEAR/3 (carcinoma* OR tumor* OR tumour* OR neoplasm*)):ti,ab,kw) AND (("national cancer database" OR NCDB):ti,ab,kw OR ("Surveillance Epidemiology" NEAR/1 "End Results"):ti,ab,kw OR (SEER NEAR/3 (data OR database OR program OR collection OR statistic* OR software)):ti,ab,kw)

<u>ClinicalTrials.gov</u>

Date Searched: 2/4/2022

Number of Results: 9

Full Search Strategy:

("Neuroendocrine neoplasms" OR "neuroendocrine carcinoma") AND ("national cancer database" OR "Surveillance Epidemiology End Results")

Table S1. Articles with insufficient information to assess nomenclature use

Citation Title	Journal	Authors + year	DOI
The role of oncologic resection and enucleation for small pancreatic neuroendocrine tumors	HPB	Altimari et al. 2021	10.1016/j.hpb.2021.03.005

Prognostic Value of Lymph Node Status and Extent of Lymphadenectomy in Pancreatic Neuroendocrine Tumors Confined To and Extending Beyond the Pancreas	Journal of Gastrointestinal Surgery	Conrad et al. 2016	10.1007/s11605-016-3243-7
Nomogram for individually predicting overall survival in rectal neuroendocrine tumours	BMC Cancer	Feng et al. 2020	10.1186/s12885-020-07328-9
Palliative resection of the primary tumor in 442 metastasized neuroendocrine tumors of the pancreas: a population-based, propensity score-matched survival analysis	Langenbeck's Archives of Surgery	Hüttner et al. 2015	10.1007/s00423-015-1323-x
Revised Staging Classification Improves Outcome Prediction for Small Intestinal Neuroendocrine Tumors	Journal of Clinical Oncology	Kim et al. 2013	10.1200/jco.2013.51.1477
Prognostic Significance of Lymph Node Metastases in Small Intestinal Neuroendocrine Tumors	Neuroendocrinology	Kim et al. 2015	10.1159/000371807
Resection of At-Risk Mesenteric Lymph Nodes Is Associated with Improved Survival in Patients with Small Bowel Neuroendocrine Tumors	World Journal of Surgery	Landry et al. 2013	10.1007/s00268-013-1918-8
Incidence Trends of Gastroenteropancreatic Neuroendocrine Tumors in the United States	Clinical Gastroenterology and Hepatology	Lee et al. 2019	10.1016/j.cgh.2018.12.017
Assessment of the American Joint Commission on Cancer 8th Edition Staging System for Patients with Pancreatic Neuroendocrine Tumors: A Surveillance, Epidemiology, and End Results analysis	Cancer Medicine	Li et al. 2018	10.1002/cam4.1336
Surgical management of rectal carcinoids: trends and outcomes from the Surveillance, Epidemiology, and End Results database (1988 to 2012)	The American Journal of Surgery	McConnell 2016	10.1016/j.amjsurg.2016.01.008
Gastrointestinal carcinoid: epidemiological and survival evidence from a large population-based study (n = 25 531)	Annals of Oncology	Mocellin and Nitti 2013	10.1093/annonc/mdt377
Novel nomogram combining depth of invasion and size can accurately predict the risk for regional nodal metastases for appendiceal neuroendocrine tumors (A-NET)	Journal of Surgical Oncology	Mosquera et al. 2017	10.1002/jso.24714

Optimal Lymphadenectomy in Small Bowel Neuroendocrine Tumors: Analysis of the NCDB	Journal of Gastrointestinal Surgery	Motz et al. 2018	10.1007/s11605-017-3524-9
Costs of Cancer Care for Elderly Patients with Neuroendocrine Tumors	PharmacoEconomics	Shen et al. 2018	10.1007/s40273-018-0656-z
Trends of Incidence and Survival of Gastrointestinal Neuroendocrine Tumors in the United States: A Seer Analysis	Journal of Cancer	Tsikitis et al. 2012	10.7150/jca.4502
Deaths of colon neuroendocrine tumors are associated with increasing metastatic lymph nodes and lymph node ratio	Journal of Gastrointestinal Oncology	Xiao et al. 2020	10.21037/jgo-20-444
Indications for Surgical Resection in Low-Grade Pancreatic Neuroendocrine Tumors	The American Surgeon	Fitzgerald et al. 2016	10.1177/000313481608200834
Facility type and size-stratified analysis of management patterns and outcomes of patients with localized non-functional pancreatic neuroendocrine tumors	HPB	Hue et al. 2022	10.1016/j.hpb.2021.07.008
Incidence of Additional Primary Malignancies in Patients with Pancreatic and Gastrointestinal Neuroendocrine Tumors	Annals of Surgical Oncology	Kauffmann et al. 2014	10.1245/s10434-014-3774-7
Elevated prevalence of nodal positivity in carcinoid tumours of the appendix smaller than 2 cm has a negative impact on overall survival	Colorectal Disease	Skancke et al. 2020	10.1111/codi.15287
The Epidemiology of Neuroendocrine Tumors in Taiwan: A Nation-Wide Cancer Registry-Based Study	PLoS ONE	Tsai et al. 2013	10.1371/journal.pone.0062487
Pancreatic islet cell tumors in adolescents and young adults	Journal of Pediatric Surgery	Waters et al. 2019	10.1016/j.jpedsurg.2019.01.060
Tumor Size and Depth Predict Rate of Lymph Node Metastasis in Colon Carcinoids and Can Be Used to Select Patients for Endoscopic Resection	Journal of Gastrointestinal Surgery	Natour et al. 2012	10.1007/s11605-011-1786-1
Malignant Neuroendocrine Tumors: Incidence and Outcomes in Pediatric Patients	European Journal of Pediatric Surgery	Allan et al. 2013	10.1055/s-0033-1333643
Racial and Socioeconomic Disparities in Malignant Carcinoid Cancer Cause Specific Survival: Analysis of the Surveillance, Epidemiology and End Results National Cancer Registry	Asian Pacific Journal of Cancer Prevention	Cheung 2013	10.7314/apjcp.2013.14.12.7117
The Prognostic Significance of Lymph Node Ratio in Carcinoid Tumors	Pancreas	Flatow et al. 2016	10.1097/mpa.0000000000000476

Propensity adjusted appraisal of the surgical strategy for appendiceal carcinoids	Techniques in Coloproctology	Ciarrocchi et al. 2015	10.1007/s10151-014-1249-2
Global burden of neuroendocrine tumors and changing incidence in Kentucky	Oncotarget	Chauhan et al. 2018	10.18632/oncotarget.24983
Surgery Versus Surveillance for Well-Differentiated, Nonfunctional Pancreatic Neuroendocrine Tumors: An 11-Year Analysis of the National Cancer Database	The Oncologist	Assi et al. 2020	10.1634/theoncologist.2019-0466

Table S2. Articles consistent with WHO nomenclature

**Citation Title**	**Journal**	**Authors + year**	**DOI**
Increasing Incidence of Duodenal Neuroendocrine Tumors: Incidental Discovery of Indolent Disease?	Surgery	Fitzgerald et al. 2015	10.1016/j.surg.2015.03.042
Distinct clinicopathological and prognostic features of insulinoma with synchronous distant metastasis	Pancreatology	Gao et al. 2019	10.1016/j.pan.2019.02.011
Mesenteric Lymphadenectomy in Well-Differentiated Appendiceal Neuroendocrine Tumors	Diseases of the Colon & Rectum	Raoof et al. 2017	10.1097/dcr.0000000000000852
Ampullary Neuroendocrine Tumors: Insight into a Rare Histology	Annals of Surgical Oncology	Ruff et al. 2021	10.1245/s10434-021-10371-w
Primary Tumor Site Affects Survival in Patients with Gastroenteropancreatic and Neuroendocrine Liver Metastases	International Journal of Endocrinology	Tierney et al. 2019	10.1155/2019/9871319
A Survival Analysis of Patients with Localized, Asymptomatic Pancreatic Neuroendocrine Tumors: No Surgical Survival Benefit when Examining Appropriately Selected Outcomes	Journal of Gastrointestinal Surgery	Powers et al. 2020	10.1007/s11605-019-04433-4
Octreotide Long-Acting Repeatable Among Elderly Patients with Neuroendocrine Tumors: A Survival Analysis of SEER-Medicare Data	Cancer Epidemiology, Biomarkers & Prevention	Shen et al. 2015	10.1158/1055-9965.epi-15-0336
The impact of surgery for metastatic pancreatic neuroendocrine tumor: a contemporary evaluation matching for chromogranin A level	HPB	Concors et al. 2020	10.1016/j.hpb.2019.05.011
Neuroendocrine Tumors of Meckel's Diverticula: Rare but Fare Well	The American Surgeon	de Andrade et al. 2019	10.1177/000313481908501010
Role of Lymph Node Resection and Histopathological Evaluation in Accurate Staging of Nonfunctional Pancreatic	Journal of Gastrointestinal Surgery	Ding et al. 2021	10.1007/s11605-020-04521-w

Neuroendocrine Tumors: How Many Are Enough?			
Carcinoid Syndrome and Costs of Care During the First Year After Diagnosis of Neuroendocrine Tumors Among Elderly Patients	The Oncologist	Shen et al. 2017	10.1634/theoncologist.2017-0149
Octreotide long-acting repeatable use among elderly patients with carcinoid syndrome and survival outcomes: A population-based analysis	Cancer	Shen et al. 2014	10.1002/cncr.28653
Relationship between Primary Tumor Resection for Metastatic Small Intestine Neuroendocrine Tumors and Survival: A Propensity Score-Matched Analysis	Journal of Investigative Surgery	Yan et al. 2021	10.1080/08941939.2021.2024306
Endoscopic and surgical treatment of T1N0M0 colorectal neuroendocrine tumors: a population-based comparative study	Surgical Endoscopy	Zhu et al. 2021	10.1007/s00464-021-08535-6
Outcomes of Lymph Node Dissection for Non-metastatic Pancreatic Neuroendocrine Tumors: A Propensity Score-Weighted Analysis of the National Cancer Database	Annals of Surgical Oncology	Mao et al. 2019	10.1245/s10434-019-07506-5
Intrinsic Contact Between T and N Classifications in Resected Well-Moderately Differentiated Locoregional Pancreatic Neuroendocrine Neoplasms	Annals of Surgical Oncology	Xu et al. 2017	10.1245/s10434-017-6289-1
A Modified Duodenal Neuroendocrine Tumor Staging Schema Better Defines the Risk of Lymph Node Metastasis and Disease-Free Survival	The American Surgeon	Kachare et al. 2014	10.1177/000313481408000836

Table S3. Articles that include poorly and undifferentiated neoplasms as neuroendocrine tumors

Citation Title	Journal	Authors + year	DOI	% poorly /undiff
Distinct Clinical Characteristics in Young-Onset Pancreatic Neuroendocrine Tumor	Cancers	Goksu et al. 2020	10.3390/cancers12092501	4.3
Geographic and demographic features of neuroendocrine tumors in the United	Cancer	Gosain et al. 2020	10.1002/cncr.32607	N/a

States of America: A population-based study				
Lymph Node Metastasis in the Prognosis of Gastroenteropancreatic Neuroendocrine Tumors	Pancreas	Martin et al. 2017	10.1097/mpa.0000000000000921	N/a
Anatomic site as prognostic marker of pancreatic neuroendocrine tumors: a cohort study	European Journal of Endocrinology	Badarna et al. 2019	10.1530/eje-19-0332	11.8
Effect of treatment center volume on outcomes in gastroenteropancreatic neuroendocrine tumor patients	BMC Cancer	Baeg et al. 2021	10.1186/s12885-021-07868-8	42.3
Racial disparities in operative management of localized, non-functional pancreatic neuroendocrine tumors in surgically fit patients	HPB	Bingmer et al. 2022	10.1016/j.hpb.2021.06.418	8.2
Life expectancy in pancreatic neuroendocrine cancer	Clinics and Research in Hepatology and Gastroenterology	Brooks et al. 2019	10.1016/j.clinre.2018.08.005	20.5
Rectal NETs and rectosigmoid junction NETs may need to be treated differently	Cancer Medicine	Cai et al. 2020	10.1002/cam4.2779	0.7
Neuroendocrine Tumors of the Colon and Rectum: Prognostic Relevance and Comparative Performance of Current Staging Systems	Annals of Surgical Oncology	Chagpar et al. 2013	10.1245/s10434-012-2746-z	56.3
Preoperative serum chromogranin-a is predictive of survival in locoregional jejuno-ileal small bowel neuroendocrine tumors	Surgery	Chatani et al. 2021	10.1016/j.surg.2021.01.048	1.1
Exploration of the Exact Prognostic Significance of Lymphatic Metastasis in Jejunoileal Neuroendocrine Tumors	Annals of Surgical Oncology	Chen et al. 2018	10.1245/s10434-018-6511-9	1.8
Predicting Survival of Patients With Rectal Neuroendocrine Tumors Using Machine Learning: A SEER-Based Population Study	Frontiers in Surgery	Cheng et al. 2021	10.3389/fsurg.2021.745220	15.0
Long-term Outcomes of Gastroenteropancreatic Neuroendocrine Tumors	Pancreas	Chi et al. 2018	10.1097/mpa.0000000000001005	17.6
Operative resection in early stage pancreatic neuroendocrine tumors in the United States: Are we over- or undertreating patients?	Surgery	Chivukula et al. 2020	10.1016/j.surg.2019.04.061	2.2

Predictors of Metastases in Rectal Neuroendocrine Tumors: Results of a National Cohort Study	Diseases of the Colon & Rectum	Concors et al. 2018	10.1097/dcr.0000000000001243	9.7
Neuroendocrine Tumors in Meckel's Diverticulum: Recommendation for Lymphadenectomy Regardless of Tumor Size Based on the NCDB Experience	Journal of Gastrointestinal Surgery	Dogeas et al. 2019	10.1007/s11605-018-04096-7	0.7
New lymph node staging for rectal neuroendocrine tumors	Journal of Surgical Oncology	Fields et al. 2019	10.1002/jso.25307	26.5
Colon Neuroendocrine Tumors: A New Lymph Node Staging Classification	Annals of Surgical Oncology	Fields et al. 2019	10.1245/s10434-019-07327-6	35.7
Local Excision Versus Radical Resection for 1- to 2-cm Neuroendocrine Tumors of the Rectum: A National Cancer Database Analysis	Diseases of the Colon & Rectum	Fields et al. 2019	10.1097/dcr.0000000000001210	0.4
Incidence of Lymph Node Metastases and Impact of Radical Surgery for Duodenal Neuroendocrine Tumors	Journal of Surgical Research	Fujii et al. 2021	10.1016/j.jss.2021.06.085	0.7
A Lymph Node Ratio–Based Staging Model Is Superior to the Current Staging System for Pancreatic Neuroendocrine Tumors	The Journal of Clinical Endocrinology & Metabolism	Gaitanidis et al. 2018	10.1210/jc.2017-01791	11.1
Duodenal neuroendocrine tumors: Somewhere between the pancreas and small bowel?	Journal of Surgical Oncology	Gamboa et al. 2019	10.1002/jso.25731	2.5
A novel preoperative risk score to predict lymph node positivity for rectal neuroendocrine tumors: An NCDB analysis to guide operative technique	Journal of Surgical Oncology	Gamboa et al. 2019	10.1002/jso.25679	31.9
Nomogram for predicting survival of patients with metastatic nonfunctioning pancreatic neuroendocrine tumors	Medicine	Ge et al. 2021	10.1097/md.0000000000026347	18.4
Racial/Ethnic Disparities and Survival Characteristics in Non-Pancreatic Gastrointestinal Tract Neuroendocrine Tumors	Cancers	Goksu et al. 2020	10.3390/cancers12102990	14.0
The impact of surgery in metastatic pancreatic neuroendocrine tumors: a competing risk analysis	Endocrine Connections	He et al. 2019	10.1530/ec-18-0485	44.3

Varying malignant potential of appendiceal neuroendocrine tumors: Importance of histologic subtype	Journal of Surgical Oncology	Hsu et al. 2013	10.1002/jso.23205	6.1
Size Criteria Is Not Sufficient in Selecting Patients for Local Excision Versus Radical Excision for Rectal Neuroendocrine Tumors >2 cm: A National Cancer Database Analysis	Diseases of the Colon & Rectum	Izquierdo et al. 2021	10.1097/dcr.0000000000001888	10.0
Prognostic Performance of Different Lymph Node Staging Systems in Patients With Small Bowel Neuroendocrine Tumors	Frontiers in Endocrinology	Jiang et al. 2020	10.3389/fendo.2020.00402	3.6
Racial Differences in Gastroenteropancreatic Neuroendocrine Tumor Treatment and Survival in the United States	Pancreas	Kessel et al. 2021	10.1097/mpa.0000000000001707	15.2
Population-Level Analysis of Pancreatic Neuroendocrine Tumors 2 cm or Less in Size	Annals of Surgical Oncology	Kuo and Salem 2013	10.1245/s10434-013-3005-7	8.0
Pancreatic Neuroendocrine Tumors	Pancreas	Lee et al. 2022	10.1097/mpa.0000000000002014	10.2
Using a Nomogram to Preoperatively Predict Distant Metastasis of Pancreatic Neuroendocrine Tumor in Elderly Patients	Chinese Medical Sciences Journal	Li et al. 2021	10.24920/003722	22.7
Clinicopathological features and prognosis factors for survival in elderly patients with pancreatic neuroendocrine tumor	Medicine	Li et al. 2019	10.1097/md.0000000000014576	12.9
Nomograms predict survival outcomes for distant metastatic pancreatic neuroendocrine tumor	Medicine	Li et al. 2020	10.1097/md.0000000000019593	22.8
Impact of a Prior Nonpancreatic Malignancy on Survival Outcomes of Patients With Stage IV Pancreatic Neuroendocrine Tumor	Pancreas	Li et al. 2020	10.1097/mpa.0000000000001630	32.6
Prognostic nomogram based on the metastatic lymph node ratio for gastric neuroendocrine tumour: SEER database analysis	ESMO Open	Li et al. 2020	10.1136/esmoopen-2019-000632	42.3
The eighth version of American Joint Committee on Cancer nodal classification for high grade pancreatic neuroendocrine tumor should be generalized for the whole population with this disease	Medicine	Li et al. 2020	10.1097/md.0000000000022089	8.3

Surgical resection of primary tumor improves survival of pancreatic neuroendocrine tumor with liver metastases	Oncotarget	Tao et al. 2017	10.18632/oncotarget.19523	10.3
Prognosis of patients with neuroendocrine tumor: a SEER database analysis	Cancer Management and Research	Man et al. 2018	10.2147/cmar.s174907	27.1
Association between somatostatin analogues and diabetes mellitus in gastroenteropancreatic neuroendocrine tumor patients: A Surveillance, Epidemiology, and End Results-Medicare analysis of 5235 patients	Cancer Reports	Ni et al. 2021	10.1002/cnr2.1387	30.7
Primary Tumor Resection Offers Survival Benefit in Patients with Metastatic Midgut Neuroendocrine Tumors	Annals of Surgical Oncology	Polcz et al. 2020	10.1245/s10434-020-08602-7	18.8
Clinical Outcomes for Neuroendocrine Tumors of the Duodenum and Ampulla of Vater:A Population-Based Study	Journal of Gastrointestinal Surgery	Randle et al. 2014	10.1007/s11605-013-2365-4	6.6
Trends in the Number of Lymph Nodes Evaluated Among Patients with Pancreatic Neuroendocrine Tumors in the United States: A Multi-Institutional and National Database Analysis	Annals of Surgical Oncology	Sahara et al. 2020	10.1245/s10434-019-08120-1	2.5
Surgical Resection Provides an Overall Survival Benefit for Patients with Small Pancreatic Neuroendocrine Tumors	Journal of Gastrointestinal Surgery	Sharpe et al. 2015	10.1007/s11605-014-2615-0	12.2
Multiple Machine Learnings Revealed Similar Predictive Accuracy for Prognosis of PNETs from the Surveillance, Epidemiology, and End Result Database	Journal of Cancer	Song et al. 2018	10.7150/jca.26649	32.2
Survival Benefit of Primary Tumor Resection Among Elderly Patients with Pancreatic Neuroendocrine Tumors	World Journal of Surgery	Toyoda et al. 2021	10.1007/s00268-021-06281-3	8.0
It's more than just cancer biology: Health disparities in patients with pancreatic neuroendocrine tumors	Journal of Surgical Oncology	Underwood et al. 2021	10.1002/jso.26667	12.4
Does Prior Cancer Have an Influence on the Survival Outcomes of Patients with Localized Pancreatic Neuroendocrine Tumors?	Chinese Medical Sciences Journal	Wang et al. 2021	10.24920/003803	1.5

Risk Factors of Lymph Node Metastasis in Patients with Pancreatic Neuroendocrine Tumors (PNETs)	Gastroenterology Research and Practice	Wang et al. 2020	10.1155/2020/1946156	7.9
Non-functional pancreatic neuroendocrine tumours: emerging trends in incidence and mortality	BMC Cancer	Wu et al. 2019	10.1186/s12885-019-5543-2	15.1
Survival outcomes and surgical intervention of small intestinal neuroendocrine tumors: a population based retrospective study	Oncotarget	Wu et al. 2017	10.18632/oncotarget.13632	3.3
Role of Removed Lymph Nodes on the Prognosis of M0 Small-Bowel Neuroendocrine Tumors: a Propensity Score Matching Analysis from SEER Database	Journal of Gastrointestinal Surgery	Xie et al. 2021	10.1007/s11605-021-04994-3	1.1
Palliative Resection of Primary Tumor in Metastatic Nonfunctioning Pancreatic Neuroendocrine Tumors	Journal of Surgical Research	Ye et al. 2019	10.1016/j.jss.2019.04.002	16.4
New Nodal Staging for Primary Pancreatic Neuroendocrine Tumors	Annals of Surgery	Zhang et al. 2021	10.1097/sla.0000000000003478	2.6
Local excision versus radical resection in patients with rectal neuroendocrine tumours: a propensity score match analysis	ANZ Journal of Surgery	Zhao et al. 2020	10.1111/ans.16221	0.4
Outcomes for a Large Cohort of Patients with Rectal Neuroendocrine Tumors: an Analysis of the National Cancer Database	Journal of Gastrointestinal Surgery	Zhao et al. 2021	10.1007/s11605-020-04525-6	5.8
Marital status is an independent prognostic factor for pancreatic neuroendocrine tumors patients: An analysis of the Surveillance, Epidemiology, and End Results (SEER) database	Clinics and Research in Hepatology and Gastroenterology	Zhou et al. 2017	10.1016/j.clinre.2017.02.008	17.4
Lymphovascular Invasion Is Associated with Lymph Node Involvement in Small Appendiceal Neuroendocrine Tumors	Annals of Surgical Oncology	Blakely et al. 2019	10.1245/s10434-019-07637-9	46.2
Comparable survival benefit of local excision versus radical resection for 10- to 20-mm rectal neuroendocrine tumors	European Journal of Surgical Oncology	Chen et al. 2022	10.1016/j.ejso.2021.10.029	N/a
Importance of Lymph Node Involvement in Pancreatic Neuroendocrine Tumors: Impact on Survival and Implications for Surgical Resection	Journal of Gastrointestinal Surgery	Curran et al. 2015	10.1007/s11605-014-2624-z	13.9

Trends in the Incidence, Prevalence, and Survival Outcomes in Patients With Neuroendocrine Tumors in the United States	JAMA Oncology	Dasari et al. 2017	10.1001/jamaoncol.2017.0589	32.6
Is There a Role for Surgery in Patients with Neuroendocrine Tumors of the Esophagus? A Contemporary View from the NCDB	Annals of Surgical Oncology	Erdem et al. 2020	10.1245/s10434-019-07847-1	94.0
Predicting Aggressive Behavior in Nonfunctional Pancreatic Neuroendocrine Tumors With Emphasis on Tumor Size Significance and Survival Trends: A Population-Based Analysis of 1787 Patients	The American Surgeon	Fathi et al. 2020	10.1177/0003134820919730	17.4
Predicting Survival and Response to Treatment in Gastroesophageal Neuroendocrine Tumors: An Analysis of the National Cancer Database	Annals of Surgical Oncology	Gray et al. 2018	10.1245/s10434-018-6389-6	14.9
A Predictive Nomogram for Small Intestine Neuroendocrine Tumors	Pancreas	Kelly et al. 2020	10.1097/mpa.0000000000001526	2.6
Malignant-functioning neuroendocrine tumors of the pancreas: A survival analysis	Surgery	Keutgen et al. 2016	10.1016/j.surg.2015.11.010	18.6
Resection of primary tumor site is associated with prolonged survival in metastatic nonfunctioning pancreatic neuroendocrine tumors	Surgery	Keutgen et al. 2016	10.1016/j.surg.2015.05.042	38.7
Distinct Prognostic Factors in Sporadic and Multiple Endocrine Neoplasia Type 1-Related Pancreatic Neuroendocrine Tumors	Hormone and Metabolic Research	Kfir et al. 2021	10.1055/a-1464-1276	10.5
Clinicopathological Characteristics and Survival Outcomes of Primary Hepatic Neuroendocrine Tumor: A Surveillance, Epidemiology, and End Results (SEER) Population-Based Study	Medical Science Monitor	Li et al. 2020	10.12659/msm.923375	46.7
Competing risks and cause-specific mortality in patients with pancreatic neuroendocrine tumors	European Journal of Gastroenterology & Hepatology	Li et al. 2019	10.1097/meg.0000000000001350	10.9
Impact of tumour size on metastasis and survival in patients with pancreatic neuroendocrine tumours (PNETs): A population based study	Journal of Cancer	Liu et al. 2019	10.7150/jca.27779	16.2

Competing Mortality in Patients With Neuroendocrine Tumors	American Journal of Clinical Oncology	Low et al. 2019	10.1097/coc.000000000000575	7.0
Modified Staging Classification for Pancreatic Neuroendocrine Tumors on the Basis of the American Joint Committee on Cancer and European Neuroendocrine Tumor Society Systems	Journal of Clinical Oncology	Luo et al. 2017	10.1200/jco.2016.67.8193	11.0
Head and body/tail pancreatic neuroendocrine tumors have different biological characteristics and clinical outcomes	Journal of Cancer Research and Clinical Oncology	Mei et al. 2020	10.1007/s00432-020-03303-w	11.6
Development and Validation of a Nomogram for Predicting Overall Survival in Pancreatic NeuroendocrineTumors	Translational Oncology	Miao et al. 2018	10.1016/j.tranon.2018.06.012	9.2
Reassessment of the Current American Joint Committee on Cancer Staging System for Pancreatic Neuroendocrine Tumors	Journal of the American College of Surgeons	Qadan et al. 2014	10.1016/j.jamcollsurg.2013.11.001	14.6
Epidemiologic trends in neuroendocrine tumors: An examination of incidence rates and survival of specific patient subgroups over the past 20 years	Seminars in Oncology	Sackstein et al. 2018	10.1053/j.seminoncol.2018.07.001	31.1
Survival trends of metastatic small intestinal neuroendocrine tumor: a population-based analysis of SEER database	Journal of Gastrointestinal Oncology	Shah et al. 2019	10.21037/jgo.2019.05.02	8.0
Clinical, pathological, and demographic factors associated with development of recurrences after surgical resection in elderly patients with neuroendocrine tumors	Annals of Oncology	Shen et al. 2017	10.1093/annonc/mdx164	30.8
Pre-existing Symptoms and Healthcare Utilization Prior to Diagnosis of Neuroendocrine Tumors: A SEER-Medicare Database Study	Scientific Reports	Shen et al. 2018	10.1038/s41598-018-35340-4	20.6
Racial Differences in the Incidence and Survival of Patients With Neuroendocrine Tumors	Pancreas	Shen et al. 2019	10.1097/mpa.0000000000001431	38.3

Clinical Characteristics and Prognostic Factors of Early-Onset Pancreatic Neuroendocrine Tumors	Cancer Control	Shi and Zhou 2021	10.1177/1073274820986827	21.0
Causes of Death After Neuroendocrine Tumors Diagnosis	Pancreas	Sonbol et al. 2021	10.1097/mpa.0000000000001723	24.5
Prognostic Nomograms to Predict Overall Survival and Cancer-Specific Survival of Patients With Pancreatic Neuroendocrine Tumors	Pancreas	Song et al. 2021	10.1097/mpa.0000000000001779	10.2
Survival of Patients With Gastroenteropancreatic Neuroendocrine Tumors and Diabetes Mellitus	Pancreas	Thapi et al. 2021	10.1097/mpa.0000000000001911	34.1
Clinicopathological features and prognosis of patients with gastric neuroendocrine tumors: A population-based study	Cancer Medicine	Tian et al. 2018	10.1002/cam4.1683	21.1
Resection of primary tumor may prolong survival in metastatic gastroenteropancreatic neuroendocrine tumors	Surgery	Tierney et al. 2019	10.1016/j.surg.2018.09.006	39.1
Resection of Primary Gastrointestinal Neuroendocrine Tumor Among Patients with Non-Resected Metastases Is Associated with Improved Survival: A SEER-Medicare Analysis	Journal of Gastrointestinal Surgery	Tsilimigras et al. 2021	10.1007/s11605-020-04898-8	51.9
Clinical Prognosticators of Metastatic Potential in Patients with Small Pancreatic Neuroendocrine Tumors	Journal of Gastrointestinal Surgery	Vega et al. 2021	10.1007/s11605-021-04946-x	26.3
Metastasis Prevalence and Survival of Patients with T1-2 Gastric Neuroendocrine Tumor Treated with Endoscopic Therapy and Surgery	Digestive Diseases and Sciences	Wang et al. 2022	10.1007/s10620-021-07143-7	38.2
The prognostic analysis of different metastatic patterns in pancreatic neuroendocrine tumors patients	Medicine	Wang et al. 2019	10.1097/md.0000000000017773	6.6
Consideration of Age Is Necessary for Increasing the Accuracy of the AJCC TNM Staging System of Pancreatic Neuroendocrine Tumors	Frontiers in Oncology	Wang et al. 2019	10.3389/fonc.2019.00906	15.0
Epidemiologic Trends of and Factors Associated With Overall Survival for Patients With Gastroenteropancreatic Neuroendocrine Tumors in the United States	JAMA Network Open	Xu et al. 2021	10.1001/jamanetworkopen.2021.24750	11.4

Pancreatic neuroendocrine tumours: Grade is superior to T, N, or M status in predicting outcome and selecting patients for chemotherapy:A retrospective cohort study in the SEER database	International Journal of Surgery	Zhai et al. 2019	10.1016/j.ijsu.2019.03.006	17.3
Establishment and validation of an AJCC stage- and histologic grade-based nomogram for pancreatic neuroendocrine tumors after surgical resection	Cancer Management and Research	Zhang et al. 2019	10.2147/cmar.s200340	9.0
Prognostic Value and Clinical Predictors of Lymph Node Metastases in Pancreatic Neuroendocrine Tumors	Pancreas	Zhang et al. 2020	10.1097/mpa.0000000000001493	4.4
Racial disparities in pancreatic neuroendocrine tumors survival: a SEER study	Cancer Medicine	Zhou et al. 2017	10.1002/cam4.1220	15.3
Frequency of carcinoid syndrome at neuroendocrine tumour diagnosis: a population-based study	The Lancet Oncology	Halperin et al. 2017	10.1016/s1470-2045(17)30110-9	6.3
Prognostic and metastasis-related factors in colorectal neuroendocrine tumors: A cross-sectional study based on the Surveillance, Epidemiology and End Results	Oncology Letters	Zou et al. 2019	10.3892/ol.2019.10876	31.8
Primary Gallbladder Neuroendocrine Tumors: Insights into a Rare Histology Using a Large National Database	Annals of Surgical Oncology	Ayabe et al. 2019	10.1245/s10434-019-07440-6	73.2
A Modified T-Stage Classification for Gastric Neuroendocrine Tumors	Journal of Surgical Research	Yang et al. 2022	10.1016/j.jss.2021.10.002	22.8
The Lymph Node Ratio Optimizes Staging in Patients with Small Intestinal Neuroendocrine Tumors	Neuroendocrinology	Wu et al. 2018	10.1159/000491017	N/a
Octreotide LAR Dosage and Survival Among Elderly Patients With Distant-Stage Neuroendocrine Tumors	The Oncologist	Shen et al. 2016	10.1634/theoncologist.2015-0381	N/a
Presentation and survival of gastro-entero-pancreatic neuroendocrine tumors in young adults versus older patients	The American Journal of Surgery	Nguyen et al. 2022	10.1016/j.amjsurg.2021.08.030	N/a
Long-Term Survival with Long-Acting Somatostatin Analogues Plus Aggressive Cytoreductive Surgery in Patients with Metastatic Neuroendocrine Carcinoma	Journal of the American College of Surgeons	Deutsch et al. 2015	10.1016/j.jamcollsurg.2015.03.055	51.0

The clinical characteristics and survival associations of pancreatic neuroendocrine tumors: does age matter?	Gland Surgery	Liu et al. 2021	10.21037/gs-20-634	7.5
Lymphovascular Invasion Predicts Lymph Node Involvement in Small Pancreatic Neuroendocrine Tumors	Neuroendocrinology	Blakely et al. 2020	10.1159/000502581	2.3
Prognosis analysis of patients with pancreatic neuroendocrine tumors after surgical resection and the application of enucleation	World Journal of Surgical Oncology	Chen et al. 2021	10.1186/s12957-020-02115-z	5.6
Development of a nomogram model to predict survival outcomes in patients with primary hepatic neuroendocrine tumors based on SEER database	BMC Cancer	Zhang et al. 2021	10.1186/s12885-021-08337-y	42.1
Pancreaticoduodenectomy and metastasectomy for metastatic pancreatic neuroendocrine tumors	Journal of Surgical Oncology	Chawla et al. 2018	10.1002/jso.25219	39.5
Practice Patterns and Guideline Non-Adherence in Surgical Management of Appendiceal Carcinoid Tumors	Journal of the American College of Surgeons	Heller et al. 2019	10.1016/j.jamcollsurg.2019.02.050	N/a
Management of 1- to 2-cm Carcinoid Tumors of the Appendix: Using the National Cancer Data Base to Address Controversies in General Surgery	Journal of the American College of Surgeons	Nussbaum et al. 2015	10.1016/j.jamcollsurg.2015.01.005	12.7
National Rise of Primary Pancreatic Carcinoid Tumors: Comparison to Functional and Nonfunctional Pancreatic Neuroendocrine Tumors	Journal of the American College of Surgeons	Kasumova et al. 2017	10.1016/j.jamcollsurg.2016.11.014	N/a
Gastric Neuroendocrine Tumors (G-Nets): Incidence, Prognosis and Recent Trend Toward Improved Survival	Cellular Physiology and Biochemistry	Yang et al. 2018	10.1159/000486915	N/a
Impact of Pancreatic Neuroendocrine Tumor on Mortality in Patients With von Hippel-Lindau Disease	Endocrine Practice	Arnon et al. 2021	10.1016/j.eprac.2021.03.005	9.0
			**Average:**	**18.7%**

Table S4. Articles reporting neuroendocrine-specific grades

**Citation Title**	**Journal**	**Authors + year**	**DOI**

Risk factors for progression of appendiceal neuroendocrine tumours: low-stage tumours <5 mm appear to be overwhelmingly indolent and may merit a separate designation	Histopathology	Noor et al. 2021	10.1111/his.14369
Midgut Neuroendocrine Tumors with Liver-only Metastases: Benefit of Primary Tumor Resection	Annals of Surgical Oncology	Gangi et al. 2020	10.1245/s10434-020-08510-w
Development and Validation of a Modified Eighth AJCC Staging System for Primary Pancreatic Neuroendocrine Tumors	Annals of Surgery	Zhang et al. 2022	10.1097/sla.0000000000004039
Revised nodal stage for pancreatic neuroendocrine tumors	Pancreatology	Luo et al. 2017	10.1016/j.pan.2017.06.003
A prognostic nomogram in patients with distant metastasis of pancreatic neuroendocrine tumors: a population-based study	Future Oncology	Cai et al. 2020	10.2217/fon-2019-0545
Lymphovascular Invasion Predicts Lymph Node Involvement in Small Pancreatic Neuroendocrine Tumors	Neuroendocrinology	Blakely et al. 2020	10.1159/000502581
Prognosis analysis of patients with pancreatic neuroendocrine tumors after surgical resection and the application of enucleation	World Journal of Surgical Oncology	Chen et al. 2021	10.1186/s12957-020-02115-z
Development of a nomogram model to predict survival outcomes in patients with primary hepatic neuroendocrine tumors based on SEER database	BMC Cancer	Zhang et al. 2021	10.1186/s12885-021-08337-y
Pancreaticoduodenectomy and metastasectomy for	Journal of Surgical Oncology	Chawla et al. 2018	10.1002/jso.25219

metastatic pancreatic neuroendocrine tumors			
Appendiceal Carcinoid Tumors: Is There a Survival Advantage to Colectomy over Appendectomy?	Journal of Gastrointestinal Surgery	Guzmán et al. 2020	10.1007/s11605-019-04306-w
Gastric Neuroendocrine Tumors (G-Nets): Incidence, Prognosis and Recent Trend Toward Improved Survival	Cellular Physiology and Biochemistry	Yang et al. 2018	10.1159/000486915
Characterization of a Novel Entity of G3 (High-grade Well-differentiated) Colorectal Neuroendocrine Tumors (NET) in the SEER Database	American Journal of Clinical Oncology	Abdel-Rahman 2021	10.1097/coc.0000000000000777
Prognostic significance of Chromogranin A in small pancreatic neuroendocrine tumors	Surgery	Raoof et al. 2019	10.1016/j.surg.2018.10.018
Impact of Pancreatic Neuroendocrine Tumor on Mortality in Patients With von Hippel-Lindau Disease	Endocrine Practice	Arnon et al. 2021	10.1016/j.eprac.2021.03.005

Table S5. Articles that use the term "carcinoid"

**Citation Title**	**Journal**	**Authors + year**	**DOI**
A Modified Duodenal Neuroendocrine Tumor Staging Schema Better Defines the Risk of Lymph Node Metastasis and Disease-free Survival	The American Surgeon	Kachare et al. 2014	10.1177/000313481408000836
Elevated prevalence of nodal positivity in carcinoid tumours of the appendix smaller than 2 cm has a negative impact on overall survival	Colorectal Disease	Skancke et al. 2020	10.1111/codi.15287
The Epidemiology of Neuroendocrine Tumors in Taiwan: A Nation-Wide Cancer Registry-Based Study	PLoS ONE	Tsai et al. 2013	10.1371/journal.pone.0062487

Pancreatic islet cell tumors in adolescents and young adults	Journal of Pediatric Surgery	Waters et al. 2019	10.1016/j.jpedsurg.2019.01.060
Tumor Size and Depth Predict Rate of Lymph Node Metastasis in Colon Carcinoids and Can Be Used to Select Patients for Endoscopic Resection	Journal of Gastrointestinal Surgery	Natour et al. 2012	10.1007/s11605-011-1786-1
Malignant Neuroendocrine Tumors: Incidence and Outcomes in Pediatric Patients	European Journal of Pediatric Surgery	Allan et al. 2013	10.1055/s-0033-1333643
Racial and Socioeconomic Disparities in Malignant Carcinoid Cancer Cause Specific Survival: Analysis of the Surveillance, Epidemiology and End Results National Cancer Registry	Asian Pacific Journal of Cancer Prevention	Cheung 2013	10.7314/apjcp.2013.14.12.7117
The Prognostic Significance of Lymph Node Ratio in Carcinoid Tumors	Pancreas	Flatow et al. 2016	10.1097/mpa.0000000000000476
Propensity adjusted appraisal of the surgical strategy for appendiceal carcinoids	Techniques in Coloproctology	Ciarrocchi et al. 2015	10.1007/s10151-014-1249-2
Appendiceal Carcinoid Tumors: Is There a Survival Advantage to Colectomy over Appendectomy?	Journal of Gastrointestinal Surgery	Guzmán et al. 2020	10.1007/s11605-019-04306-w
Practice Patterns and Guideline Non-Adherence in Surgical Management of Appendiceal Carcinoid Tumors	Journal of the American College of Surgeons	Heller et al. 2019	10.1016/j.jamcollsurg.2019.02.050
Management of 1- to 2-cm Carcinoid Tumors of the Appendix: Using the National Cancer Data Base to Address Controversies in General Surgery	Journal of the American College of Surgeons	Nussbaum et al. 2015	10.1016/j.jamcollsurg.2015.01.005
National Rise of Primary Pancreatic Carcinoid Tumors: Comparison to Functional and	Journal of the American College of Surgeons	Kasumova et al. 2017	10.1016/j.jamcollsurg.2016.11.014

Nonfunctional Pancreatic Neuroendocrine Tumors			
Gastric Neuroendocrine Tumors (G-Nets): Incidence, Prognosis and Recent Trend Toward Improved Survival	Cellular Physiology and Biochemistry	Yang et al. 2018	10.1159/000486915
Development of a nomogram model to predict survival outcomes in patients with primary hepatic neuroendocrine tumors based on SEER database	BMC Cancer	Zhang et al. 2021	10.1186/s12885-021-08337-y
